# Hepatocyte-specific PPARγ Deletion Uncovers Role of an Antagonistic PPARγ–HNF4α Transcriptional Axis in Metabolic Dysfunction–Associated Steatotic Liver Disease Progression

**DOI:** 10.1016/j.ajpath.2026.04.015

**Published:** 2026-05-12

**Authors:** Shehnaz Bano, Matthew A. Copeland, Jia-Jun Liu, Anne Orr, John W. Stoops, Wendy M. Mars, Silvia Liu, Joseph Locker, George K. Michalopoulos, Bharat Bhushan

**Affiliations:** ∗ Department of Pathology, University of Pittsburgh School of Medicine, Pittsburgh, Pennsylvania; † Pittsburgh Liver Research Center, University of Pittsburgh, Pittsburgh, Pennsylvania; ‡ Organ Pathobiology and Therapeutics Institute, University of Pittsburgh School of Medicine, Pittsburgh, Pennsylvania; § Department of Pharmacology and Chemical Biology, University of Pittsburgh School of Medicine, Pittsburgh, Pennsylvania

## Abstract

Systemic peroxisome proliferator-activated receptor gamma (PPARγ) agonist therapies have been investigated for clinical use in metabolic dysfunction–associated steatotic liver disease (MASLD). Previous studies, however, have shown that PPARγ activation in hepatocytes worsens MASLD. Overall, the precise molecular mechanisms regulated by PPARγ in hepatocytes during MASLD remain incompletely defined. Hepatocyte-specific PPARγ knockout mice were fed a clinically relevant fast-food diet for 2 or 5 months to characterize the role of PPARγ in MASLD progression. PPARγ expression and activity were increased in the murine fast-food diet model and in hepatocytes of patients with MASLD. PPARγ deletion protected against steatosis, fibrosis, and liver injury by suppressing lipogenic, inflammatory, and fibrogenic signaling. While *de novo* fatty acid synthesis pathway was only modestly altered and compensated with disease progression, effects on lipid droplet remodeling were persistent. PPARγ deletion consistently inhibited signaling of transforming growth factor-β1, a key driver of fibrosis. Importantly, a novel PPARγ–hepatocyte nuclear factor 4α (HNF4α) regulatory axis in MASLD was identified, with increased HNF4α mRNA/protein expression and activation of its downstream networks in PPARγ-deficient livers. Integrated chromatin immunoprecipitation sequencing and transcriptomics analysis suggests that HNF4α co-regulates over 70% of PPARγ target genes during MASLD. This study provides a comprehensive temporal framework for hepatocyte PPARγ function in MASLD, and reveals an antagonistic PPARγ–HNF4α transcriptional network governing hepatic metabolism.

## Graphical abstract

**Figure:**
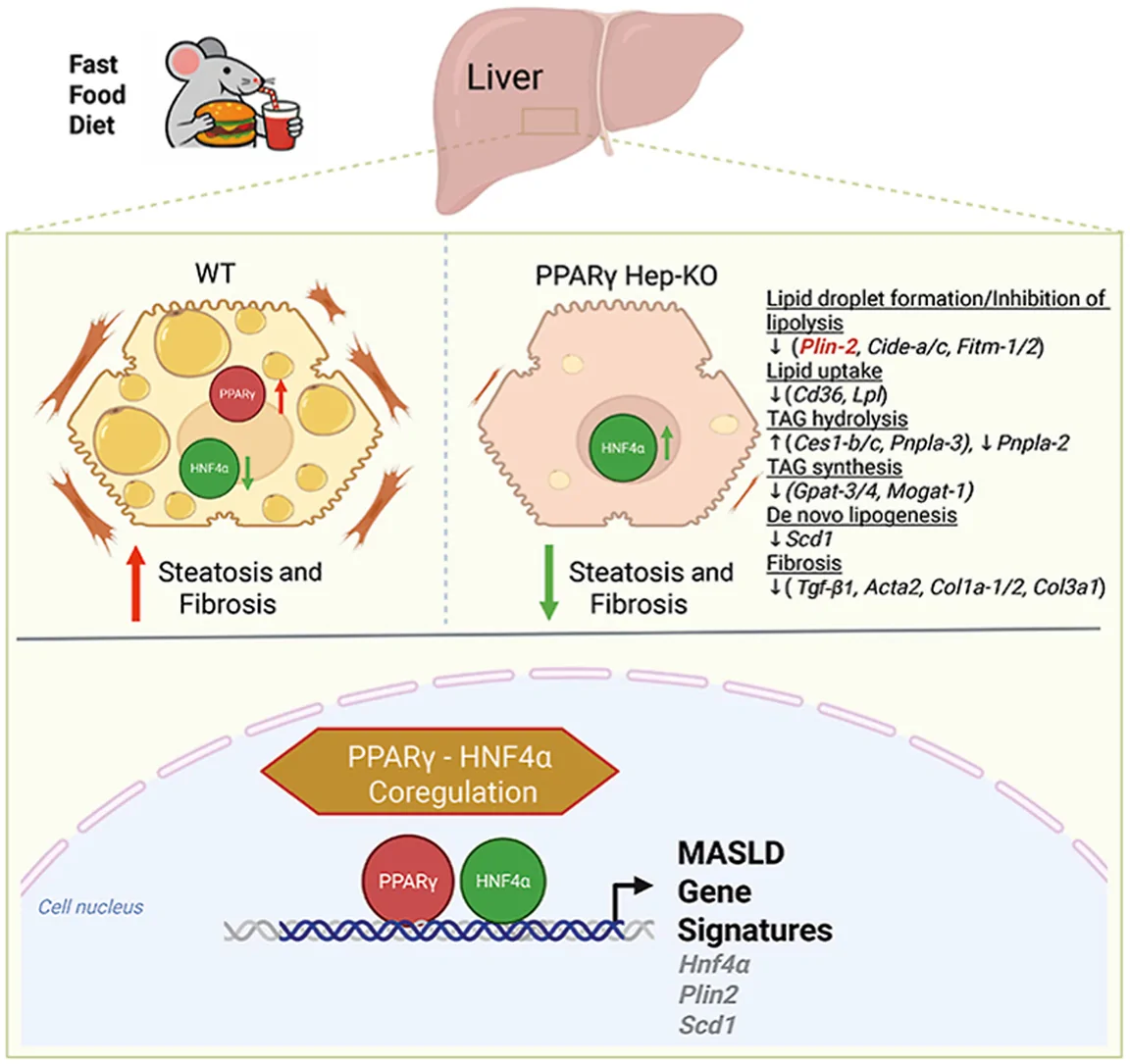

Metabolic dysfunction–associated steatotic liver disease (MASLD), formerly known as nonalcoholic fatty liver disease, is the most common chronic liver disorder worldwide, affecting nearly one-third of the global population and up to 70% of individuals with obesity or type 2 diabetes.^1^ MASLD encompasses a spectrum, from simple steatosis to metabolic dysfunction–associated steatohepatitis (MASH), characterized by hepatocyte injury, inflammation, and progressive fibrosis that can lead to cirrhosis and hepatocellular carcinoma.^2^ Despite its clinical significance, effective pharmacologic treatments remain limited, reflecting incomplete understanding of the molecular and cellular mechanisms underlying disease progression.

Peroxisome proliferator-activated receptors (PPARs) are nuclear receptors that regulate lipid and glucose metabolism, inflammation, and fibrogenesis.^3^ Among the three isoforms, PPAR alpha (PPARα), PPAR beta/delta, and PPAR gamma (PPARγ), PPARγ is the target of thiazolidinediones, insulin-sensitizing drugs with efficacy reported in MASH clinical trials.^4^, ^5^, ^6^, ^7^, ^8^ However, thiazolidinediones exhibit paradoxical effects in MASLD, improving systemic insulin sensitivity and reducing inflammation and fibrosis, yet sometimes exacerbating hepatic steatosis in preclinical models.^9^, ^10^, ^11^

This complexity highlights the need to clarify the cell-specific roles of PPARγ in the liver. PPARγ is expressed in hepatocytes, Kupffer cells, hepatic stellate cells, and infiltrating immune cells in the liver, with expression patterns varying by cell type and disease state.^12^ Hepatocytes, which constitute the majority of liver mass, express low basal levels of PPARγ, although their expression is markedly induced in obesity, insulin resistance, and MASLD.^8^^,^^13^ This induction promotes lipid accumulation and triglyceride synthesis, implicating hepatocyte PPARγ as a potential driver of liver steatosis.^9^^,^^14^^,^^15^

The dual roles of PPARγ (steatogenic in hepatocytes versus anti-inflammatory and antifibrotic in non-parenchymal cells) complicate interpretation of thiazolidinediones’ effects and underscore the importance of dissecting cell-specific functions.^11^^,^^12^ For example, PPARγ activation in hepatic stellate cells maintains quiescence and suppresses fibrogenesis, while in macrophages it promotes an anti-inflammatory phenotype.^16^^,^^17^ Conversely, hepatocyte-specific PPARγ activation enhances lipogenesis and may trigger release of damage-associated molecular patterns that exacerbate inflammation and fibrosis.^8^^,^^18^ Hepatocyte-specific PPARγ knockout mouse models have shown that loss of PPARγ in hepatocytes reduces diet-induced steatosis, inflammation, and fibrosis independently of systemic insulin sensitivity or adiposity.^8^^,^^9^^,^^11^^,^^19^ These models also show enhanced therapeutic responses to thiazolidinediones, suggesting that hepatocyte PPARγ limits their beneficial effects by promoting steatosis.^11^ Furthermore, earlier studies showed that ErbB receptor tyrosine kinases drive PPARγ induction in hepatocytes during MASLD, and suppression of PPARγ in hepatocytes contributes to striking protective effects of ErbB inhibitors in MASLD.^20^ However, the precise molecular pathways regulated by hepatocyte PPARγ in MASLD remain incompletely defined. It is critical to fully characterize these mechanisms to appreciate and harness the clinical potential of blocking hepatocyte-specific PPARγ, as the current clinical focus is mainly on systemic PPARγ activation.

To address this gap, the impact of hepatocyte-specific PPARγ deletion was investigated in a fast-food diet (FFD) mouse model that recapitulates all of the key features of human MASLD, including steatosis, inflammation, and fibrosis, in a single model.^20^^,^^21^ Using comprehensive longitudinal transcriptomic profiling, it was found that hepatocyte PPARγ deletion confers robust protection against diet-induced liver injury by suppressing key pro-lipogenic and pro-fibrotic gene networks. Although the inhibitory effects on lipid droplet formation/stabilization and fibrosis pathways and enhancement of lipolysis pathways were persistent over time, the effect on *de novo* lipogenesis (DNL) signaling was only modest and was compensated upon chronic dietary stress. Notably, this study uncovered, for the first time, that PPARγ suppresses transcription and activity of hepatocyte nuclear factor 4α (HNF4α) during MASLD, a transcription factor at the core of maintaining liver metabolic functionality. The current study further revealed that PPARγ co-regulation with HNF4α might be an important driver of global transcriptional reprogramming in MASLD.

## Materials and Methods

### Animal Models and Experimental Design

Male PPARγ^flox/flox^ mice (stock #004584; Jackson Laboratory, Bar Harbor, ME) at 8 weeks of age received a single i.p. injection of adeno-associated virus AAV8.TBG.PI.Cre.rBG (2.5 × 10^11^ viral particles per mouse) to generate hepatocyte-specific PPARγ knockout (KO) mice.^22^^,^^23^ This vector expresses Cre recombinase under the control of the hepatocyte-specific thyroxine-binding globulin (TBG) promoter. Control littermates were administered the same dose of AAV8.TBG.PI.eGFP.WPRE.bGH (2.5 × 10^11^ viral particles per mouse). Both viral preparations were obtained from Addgene (Watertown, MA; Cre vector: #107787-AAV8; eGFP vector: #105535-AAV8).

After the AAV8 injections, both the KO and control group were placed on an FFD (#TD.88137; Envigo, Indianapolis, IN), consisting of high saturated fat (21% by weight; 42% kcal from fat), cholesterol (0.2%), and sucrose (34% by weight), accompanied by fructose-glucose–enriched drinking water (18.9 g/L d-glucose and 23.1 g/L d-fructose) as previously described.^20^^,^^21^^,^^24^ Mice were maintained on this regimen for either 2 or 5 months.

### Animal Housing and Welfare

All animals were housed in an AAALAC-accredited facility at the University of Pittsburgh under a 12-hour light/dark cycle with free access to food and water. All experimental procedures were reviewed and approved by the Institutional Animal Care and Use Committee.

### Glucose and Insulin Tolerance Tests

For the glucose tolerance test, mice were fasted for 8 hours before receiving i.p. dextrose (2 g/kg in sterile phosphate-buffered saline). Blood glucose was measured at 0, 15, 30, 60, 90, and 120 minutes. For the insulin tolerance test, animals were fasted for 4 hours before i.p. injection of human regular insulin (0.5 U/kg in phosphate-buffered saline), with glucose measured at 0, 15, 30, and 45 minutes.

### Serum Biochemistry and Histology

Blood was collected for serum separation and analyzed for alanine aminotransferase, aspartate aminotransferase, cholesterol, and triglycerides. Formalin-fixed, paraffin-embedded liver sections were stained with hematoxylin and eosin or Sirius Red. Sirius Red was prepared by dissolving 0.5 g Direct Red 80 [#365548; MilliporeSigma (Sigma-Aldrich), Burlington, MA] in 1 L of saturated picric acid. Sections were deparaffinized, rehydrated, stained, rinsed in acidified water, dehydrated through graded ethanol, and cleared before mounting. Frozen sections were stained with Oil Red O using a commercial kit (#KTORO EA; StatLab Medical Products, McKinney, TX). Immunohistochemistry for α-smooth muscle actin (#19245, 1:300; Cell Signaling Technology, Danvers, MA) was performed to assess stellate cell activation. Quantitative analysis for Sirius Red staining was conducted by using ImageJ version 1.54p software (NIH, Bethesda, MD; *https://imagej.net/ij*).

### Hepatic Triglyceride Quantification

Approximately 100 mg of frozen liver tissue was homogenized in 3 mL chloroform: methanol (2:1) in glass vials. Samples were rotated at room temperature for 90 to 120 minutes, with intermittent vortexing, followed by acidification with 1 mol/L H_2_SO_4_ and centrifugation (2000 × *g*, 10 minutes). The organic phase was collected, and an aliquot (50 μL) was evaporated to dryness. Triglycerides were quantified by using the Infinity Triglyceride Reagent (Thermo Fisher Scientific, Waltham, MA) and normalized to tissue weight.

### Protein Isolation and Western Blotting

Whole-cell lysates were prepared in RIPA buffer and separated by using NuPAGE 4 to 12% Bis-Tris gels (Invitrogen, Carlsbad, CA) with MOPS running buffer, followed by transfer to polyvinylidene difluoride membranes (MilliporeSigma) in NuPAGE transfer buffer containing 10% methanol. Primary antibodies were used at 1:1000 dilution and secondary antibodies at 1:2000, unless specified otherwise. Antibodies included: GAPDH (#5174S), FASN (#3180S), ACC (#3676S), ACLY (#4332S), PPARγ (#2435S), SCD-1 (#2794S), and β-actin-HRP (#12262S) from Cell Signaling Technology; SREBP-1 (1:200; sc-13551), TGF-β1 (sc-146), and LPL (sc-373759) from Santa Cruz Biotechnology (Dallas, TX); HNF4α (1:1000; PP-H1415-00) from R&D Systems (Minneapolis, MN); and PLIN2 (#ab108323) from Abcam (Cambridge, United Kingdom).

### Retrieval and Analysis of Single-Nuclei RNA-Sequencing Data Sets from MASLD Patients

Publicly available single-nuclei RNA-sequencing data sets on patients with MASLD [MASH-associated cirrhosis (MASH-cirrhosis) stage] and healthy control individuals were obtained from the Gene Expression Omnibus database (*http://www.ncbi.nlm.nih.gov/geo*; accession number GSE256398). The data set included four patients with MASH-cirrhosis and six healthy control individuals.

Data were retrieved to analyze hepatocyte-specific gene expression changes. Differentially expressed genes (DEGs) between MASH-cirrhosis versus healthy controls (specifically in hepatocytes) exhibiting a significance threshold of *P* < 0.0001 and fold change >2.75 were selected for further Ingenuity Pathway Analysis (IPA).

### RNA Sequencing and Bioinformatics

Total RNA was extracted using the TRIzol (MilliporeSigma) method and submitted to Novogene (Sacramento, CA) for quality control, library preparation, sequencing, and alignment to the mouse reference genome (mm10) using STAR. Sequencing data are available in the Gene Expression Omnibus database (*http://www.ncbi.nlm.nih.gov/geo*; accession number GSE328680). DEGs were identified by using statistical significance criteria (*P* < 0.05) and additionally filtered by using a ≥1.3-fold change threshold for pathway analysis. Pathway and upstream regulator analyses were performed by using Ingenuity Pathways Analysis (IPA) version 8.0 (Qiagen, Redwood City, CA) and Database for Annotation, Visualization, and Integrated Discovery analysis software (DAVID Knowledgebase version 2021q4; *https://davidbioinformatics.nih.gov*, last accessed September 2025). DAVID was also used to determine enrichment in Gene Ontology terms, Kyoto Encyclopedia of Genes and Genomes pathways, and Reactome pathways, using *Mus musculus* as background reference.

### Chromatin Immunoprecipitation Sequencing Data Analysis

Chromatin immunoprecipitation sequencing (ChIP-seq) peak sets were compiled from analysis of publicly available and previously characterized ChIP-seq data sets of PPARα, HNF4α, retinoid X receptor alpha, and hepatocyte nuclear factor 1α (HNF1α) from quiescent liver.^20^^,^^25^^,^^26^ The HNF1α peak set was compiled from ArrayExpress files (*https://www.ebi.ac.uk/biostudies/arrayexpress*; accession number E-MTAB-941). Due to the unavailability of a ChIP-seq data set of PPARγ in mouse liver, PPARα was used as a surrogate marker. PPARα and PPARγ have virtually identical DNA binding,^27^ and the strength and position of their binding sites are highly concordant in mouse liver.^20^ Additional ChIP-seq data sets for HeK4me3 and H4K5ac defined ChIP-seq peak subsets that were within transcriptionally active chromatin.^26^ Peaks were also characterized to discriminate enhancers that bound HNF4α, PPARα, or a combination of both factors. Binding peaks were then linked to the nearest expressed liver gene transcription start site within 100 kb using RnaChipIntegrator.

### Chromatin Immunoprecipitation

ChIP for PPARγ and HNF4α was performed as described previously.^20^ In summary, chromatin was prepared from 120 mg of frozen liver tissue using the truChIP Chromatin Shearing Tissue Kit with Formaldehyde (catalog #520237; Covaris, Woburn, MA), and chromatin was further sheared by using the Adaptive Focused Acoustics Ultrasonicator (Covaris). Immunoprecipitation was performed with the ChIP-IT High-Sensitivity kit (catalog #53040; Active Motif, Carlsbad, CA) using 30 μg of chromatin and 10 μg of PPARγ (rabbit monoclonal antibody, #2435; Cell Signaling Technology) and HNF4α (rabbit polyclonal antibody, #sc-8987x; Santa Cruz Biotechnology) antibodies. Purified DNA after immunoprecipitation was analyzed by real-time PCR using primers designed for the strongest PPAR enhancer site (–6.3 kb) near *Hnf4a* gene, which also share a HNF4α-binding site. The HNF1α-binding site near the *Hnf4a* promoter (–0.06 kb), a region that does not bind HNF4α or PPAR, served as a negative control. Data were normalized with the input DNA.

### Statistical Analysis

Data are expressed as means ± SEM. Comparisons between two groups were performed by using the *t*-test, and multiple group comparisons by one-way analysis of variance with Tukey post hoc test. Statistical significance was defined as *P* < 0.05, with thresholds indicated as ∗*P* < 0.05, ∗∗*P* < 0.01, ∗∗*P* < 0.001, ∗∗∗∗*P* < 0.0001, and ^#^*P* < 0.00001.

## Results

### Conserved Activation of PPARγ Downstream Gene Networks in Human MASLD/MASH and Mouse FFD Model

**Figure 1:**
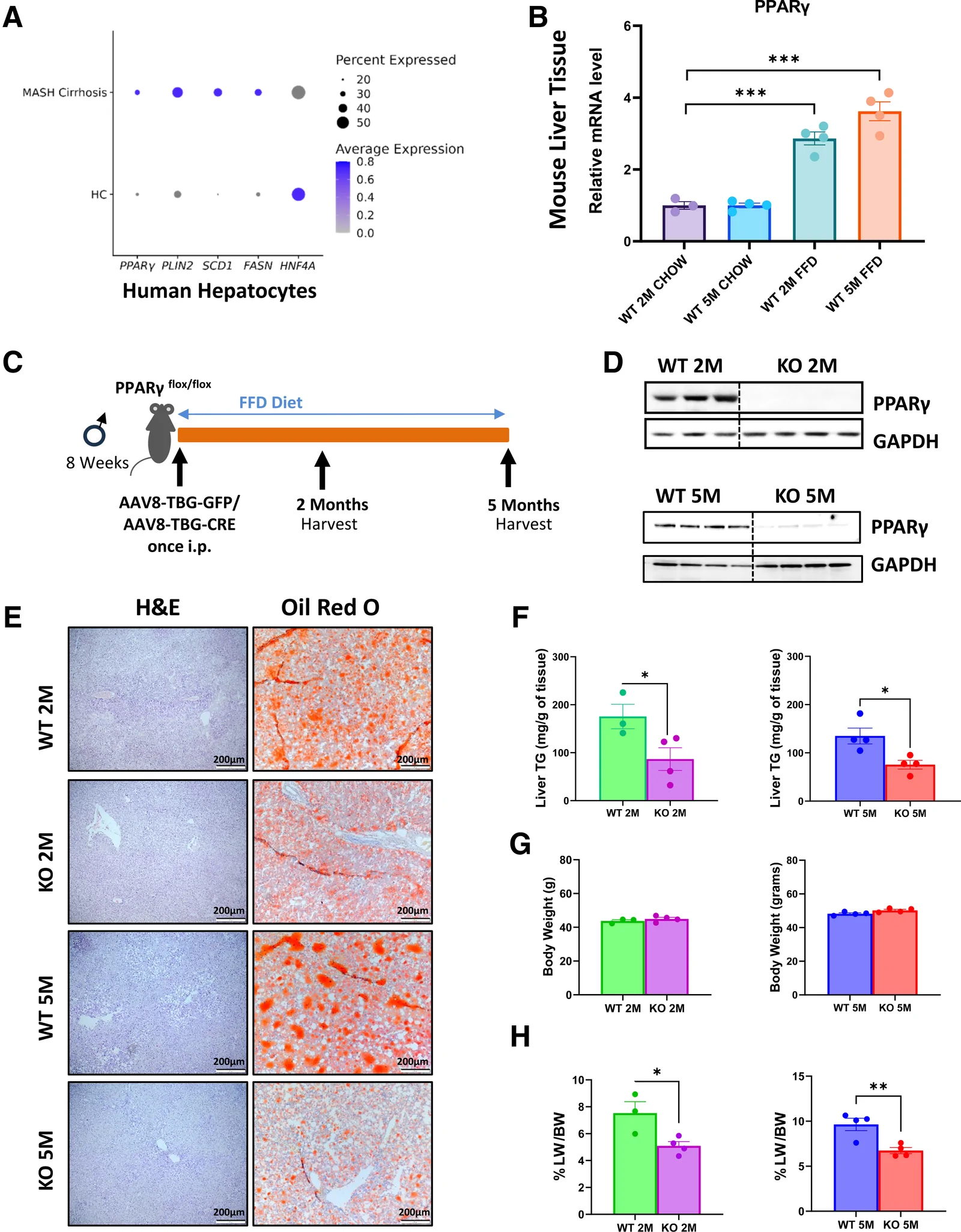
Hepatocyte-specific peroxisome proliferator-activated receptor gamma (PPARγ) deletion protects against liver steatosis in a murine fast-food diet (FFD) model of metabolic dysfunction–associated steatotic liver disease. **A:** mRNA expression of PPARγ and its target genes associated with lipogenesis (*PLIN2, SCD1,* and *FASN*) and *HNF4A* in human hepatocytes from metabolic dysfunction–associated steatohepatitis (MASH)-associated cirrhosis patients (MASH Cirrhosis) versus healthy control (HC) individuals, represented as a dot plot displaying average expression along with percentage of cells showing significant expression (identified by analysis of published single-nuclei RNA-sequencing data). **B:** PPARγ mRNA expression in liver tissue from mice fed chow and FFD for 2 months (2M) and 5 months (5M). **C:** Schematic of experimental design. PPARγ^flox/flox^ mice received a single i.p. injection of either AAV8-TBG-GFP (control) or AAV8-TBG-Cre [for hepatocyte-specific knockout (KO)] and fed an FFD for 2 or 5 months. **D:** Western blot analysis of PPARγ in liver lysates confirms efficient KO. **E:** Representative photomicrographs of Oil Red O–stained (**right panel**) and hematoxylin and eosin (H&E)–stained (**left panel**) liver sections at 2M and 5M of wild-type (WT) and KO mice. **F–H:** Bar graphs showing liver triglycerides (TG) (**F**), body weight (**G**), and liver to body weight ratio (% LW/BW) (**H**) at 2M and 5M in FFD-fed WT and KO mice. *n* = 3/4. ∗*P* < 0.05, ∗∗*P* < 0.01, ∗∗∗*P* < 0.001. Scale bar = 200 μm (**E**). GAPDH, glyceraldehyde-3-phosphate dehydrogenase.

PPARγ is a key regulator of lipid metabolism, and its role in the liver is increasingly recognized in MASLD.^28^^,^^29^ To assess the status of PPARγ signaling in hepatocytes of patients with MASLD/MASH, publicly available data sets of single-nuclei RNA sequencing from patients with MASLD (MASH-cirrhosis stage) and healthy control subjects were analyzed. Notably, average PPARγ mRNA expression in hepatocytes and percentage of hepatocytes expressing PPARγ were elevated in patients with MASH-cirrhosis compared with healthy control subjects (Figure 1A). Consistently, some of the previously reported, bona fide targets of PPARγ (verified with ChIP in the liver) involved in lipogenesis (*PLIN2, FASN,* and *SCD1*) also exhibited a similar pattern (Figure 1A).^20^ In contrast, expression of *HNF4A,* which is the master regulator of hepatocyte differentiation, was drastically reduced in these patients (Figure 1A).

Furthermore, RNA sequencing was performed in mice subjected to FFD for 2 or 5 months. Similarly to the human data set, PPARγ mRNA levels were markedly increased in livers of FFD-fed mice relative to chow-fed control mice, with expression proportional to the duration of FFD feeding (Figure 1B). Upstream regulator analysis of DEGs using IPA further revealed significant activation of PPARγ-regulated gene networks in both species (Supplemental Figures S1 and S2). Analysis of the human MASH-cirrhosis single-nuclei RNA sequencing data set revealed that the PPARγ downstream gene network was significantly up-regulated in hepatocytes of these patients compared with controls (z-score = 2.6; *P* = 2.57 × 10^–^^5^) (Supplemental Figure S1). This activation was corroborated in the FFD-fed mouse model (z-score = 4.03; *P* = 1.25 × 10^–^^17^) (Supplemental Figure S2). The increased PPARγ network activation in both human and mouse data sets underscore a conserved metabolic response during hepatic lipid accumulation and metabolic stress across species. These findings also support the translational utility of the FFD mouse model for investigating the role of PPARγ in human MASLD pathogenesis.

### Hepatocyte-Specific PPARγ Deletion Attenuates FFD-Induced Hepatic Steatosis and Liver Damage

To precisely determine the hepatocyte-specific contribution of PPARγ within the liver to diet-induced steatosis in the FFD model, hepatocyte-specific PPARγ-KO mice were generated via AAV8-TBG-Cre–mediated recombination in PPARγ^flox/flox^ animals; AAV8-TBG-GFP–treated littermates served as controls. Both groups were subjected to the FFD, enriched in fat, cholesterol, and sucrose, complemented by a high-fructose-glucose solution in drinking water (d-glucose, 18.9 g/L; d-fructose, 23.1 g/L) for durations of 2 and 5 months, to model chronic nutritional stress driving MASLD (Figure 1C).^20^^,^^21^

Western blot analysis confirmed efficient and sustained ablation of PPARγ protein in PPARγ-KO livers at both time points, validating the durability of the KO (Figure 1D). Histopathologic evaluation of hematoxylin and eosin–stained liver sections revealed preserved hepatic architecture in PPARγ-KO mice, with a marked attenuation of macrovesicular steatosis compared with control mice (Figure 1E). Consistently, Oil Red O staining showed a substantial decrease in lipid accumulation in livers of PPARγ-KO mice at both 2 and 5 months (Figure 1E). Quantitative biochemical assays further corroborated these observations, showing significantly lower hepatic triglyceride content in PPARγ-KO livers versus control livers at both time points (Figure 1F).

Despite comparable body weights between PPARγ-KO and control mice throughout the dietary intervention, PPARγ-KO animals exhibited a significant reduction in liver-to-body weight ratios relative to control mice (Figure 1, G and H), indicating a pronounced protection against FFD-induced hepatomegaly. These findings reveal hepatocyte-specific PPARγ as a critical driver of lipid accumulation and steatosis under chronic dietary excess, using the clinically relevant FFD model.

**Figure 2:**
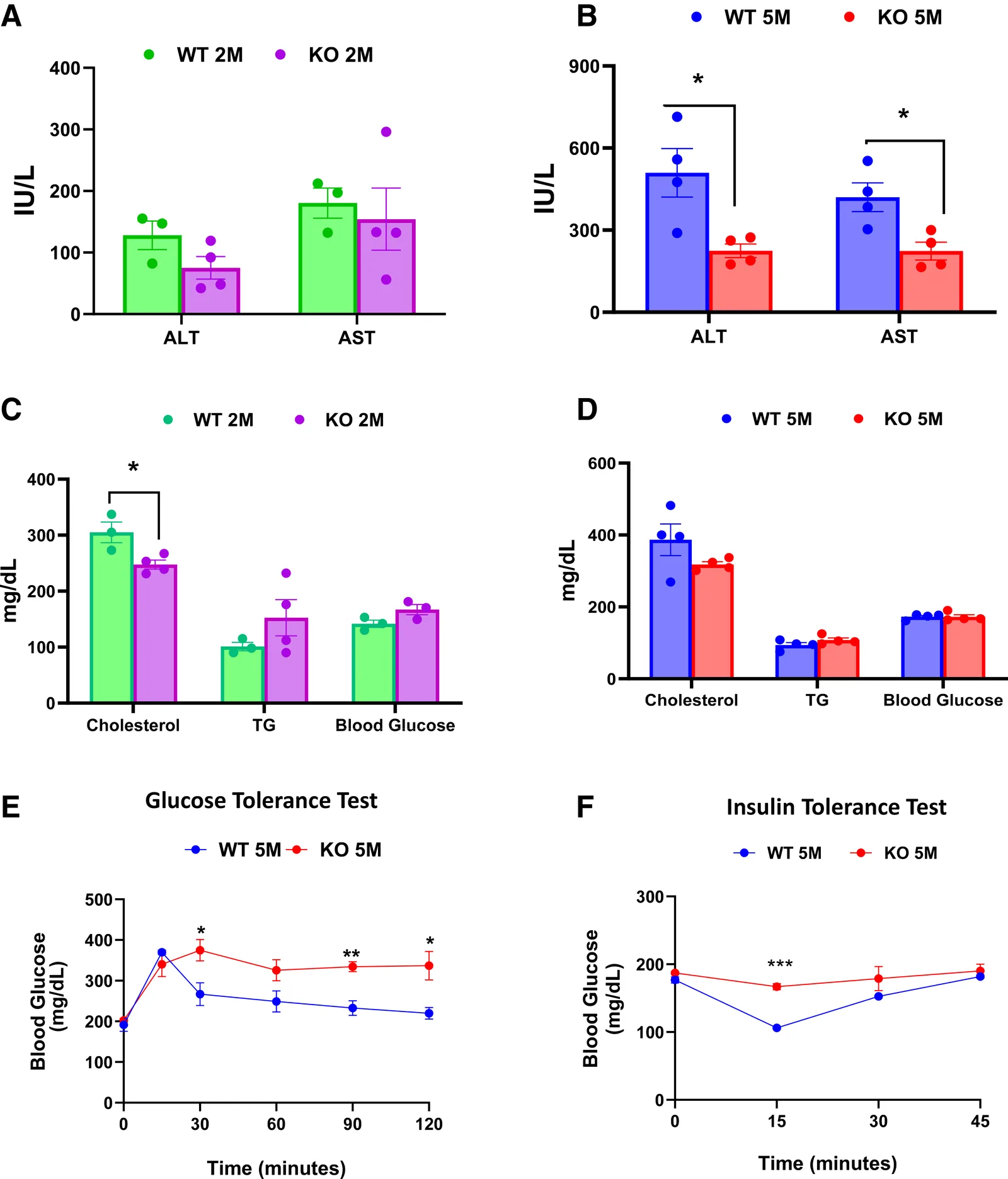
Effects of hepatocyte-specific peroxisome proliferator-activated receptor gamma (PPARγ) deletion on metabolic dysfunction–associated steatotic liver disease (MASLD)-associated serum parameters. **A–D:** Bar graphs showing alanine aminotransferase (ALT) and aspartate aminotransferase (AST) (**A** and **B**), cholesterol, triglycerides (TG), and random blood glucose levels (**C** and **D**) in serum of wild-type (WT) and knockout (KO) mice at 2 months (2M) and 5 months (5M). **E** and **F:** Glucose tolerance test (**E**) and insulin tolerance test (**F**) in WT and KO mice at 5M of fast-food diet feeding. *n* = 3/4. ∗*P* < 0.05, ∗∗*P* < 0.01, ∗∗∗*P* < 0.001.

Beyond its pronounced effect on hepatic lipid accumulation, hepatocyte-specific PPARγ ablation significantly affected indicators of liver injury and glucose homeostasis. PPARγ-KO mice showed a downward trend in serum alanine aminotransferase and aspartate aminotransferase levels at 2 months, with significant reductions at 5 months, indicating partial protection against hepatocellular injury paralleling steatosis attenuation (Figure 2, A and B). Serum cholesterol was significantly reduced at 2 months and trended lower at 5 months (Figure 2, C and D). However, glucose and insulin tolerance test results at 5 months revealed impaired glucose tolerance and insulin sensitivity in PPARγ-KO mice (Figure 2, E and F). These results underline the role of hepatocyte PPARγ in maintaining systemic glucose homeostasis under chronic dietary stress, where its absence disrupts hepatic glucose output and insulin signaling. These findings also indicate that although hepatocyte PPARγ is critical for the development of hepatic steatosis, its deletion alone is insufficient to ameliorate systemic glucose intolerance in the context of chronic FFD feeding that is consistent with previous studies.^19^

### Hepatocyte-Specific PPARγ Deletion Reprograms Key Upstream Regulators and Gene Networks Associated with MASLD in a Time-Dependent Manner

**Figure 3:**
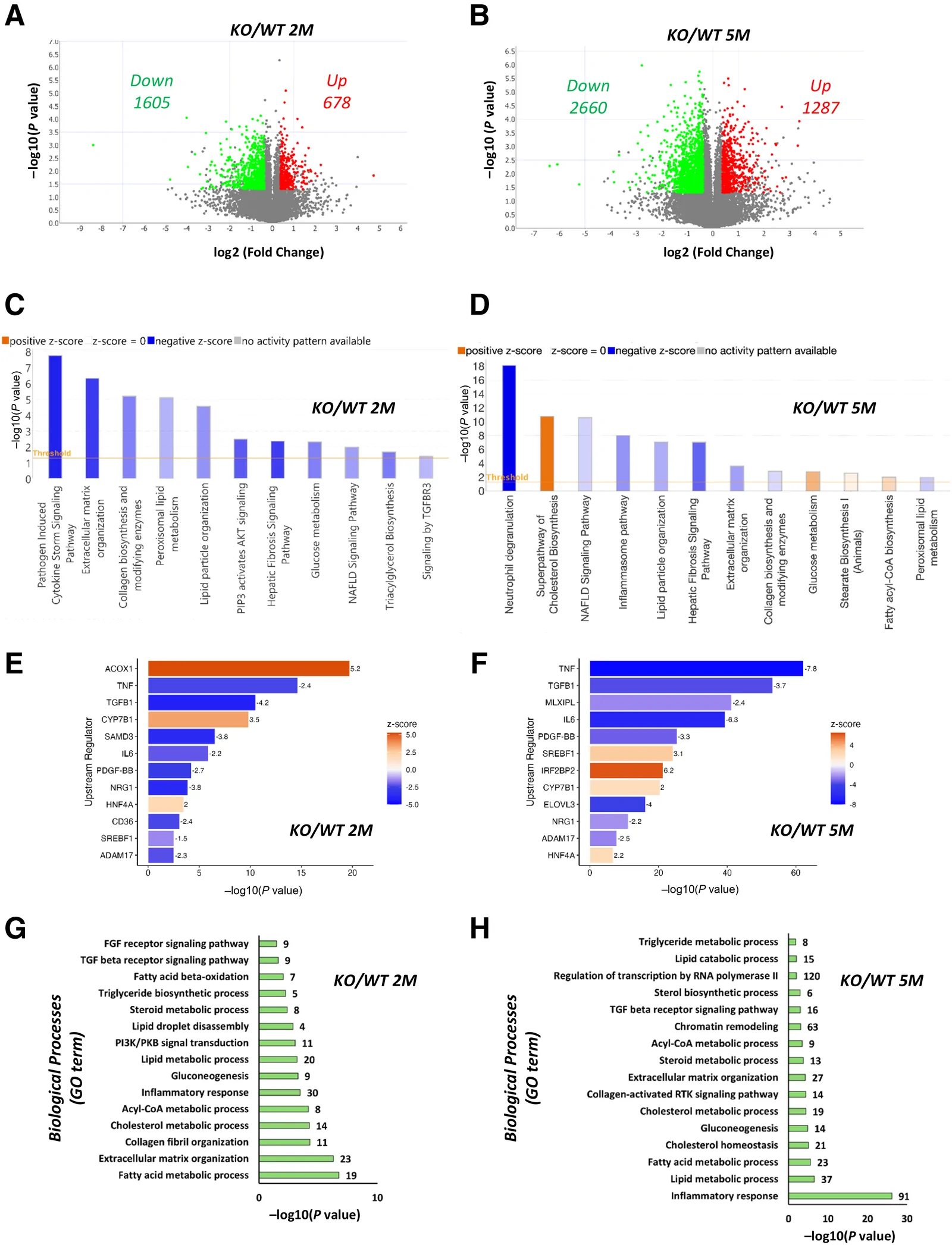
Transcriptomics analysis reveals an extensive reprogramming of hepatic metabolic gene networks in hepatocyte-specific peroxisome proliferator-activated receptor gamma-knockout (KO) mice. **A** and **B:** Volcano plots showing numbers of up-regulated and down-regulated genes at each time point [2 months (2M) (**A**) and 5 months (5M) (**B**)]. **C–H:** Canonical signaling pathway analysis (**C** and **D**) and upstream regulator analysis (**E** and **F**), using Ingenuity Pathway Analysis (IPA), and enrichment analysis using DAVID showing biological processes [Gene Ontology (GO) terms] predicted to be altered in KO versus wild-type (WT) livers at 2M (**G**) and 5M (**H**). *n* = 3.

Hepatocyte-specific PPARγ deletion induced a dynamic, time-dependent reprogramming of hepatic gene expression during MASLD progression. Transcriptomic profiling of PPARγ-KO versus wild-type livers after 2 and 5 months of FFD feeding revealed a progressive increase in DEGs, with 1605 down-regulated and 678 up-regulated genes at 2 months, rising to 2660 down-regulated and 1287 up-regulated genes at 5 months (Figure 3, A and B and Supplemental Tables S1 and S2). IPA consistently identified inhibition of canonical pathways related to hepatic fibrosis, inflammation, lipid particle organization, extracellular matrix remodeling, and MASLD signaling in PPARγ-KO livers at both time points (Figure 3, C and D). Although triacylglycerol biosynthesis was suppressed at 2 months, stearate, fatty acyl-CoA, and cholesterol biosynthesis were activated at 5 months, indicating a temporal shift in lipid metabolic regulation (Figure 3, C and D). Similarly, upstream regulator analysis revealed an early trend in suppression of the SREBF1 lipogenic network (z-score = –1.477; *P* = 3.07 × 10^–^^3^) at 2 months, but it was remarkably activated at 5 months (z-score = 3.056; *P* = 8.49 × 10^–^^25^) (Figure 3, E and F and Supplemental Figure S3). However, persistent inhibition of important inflammatory and fibrotic mediators [transforming growth factor-β1 (TGF-β1), IL-6, and tumor necrosis factor] was noted across both time points (Figure 3, E and F).

Interestingly, HNF4α, a key transcription factor for maintaining hepatic function and metabolism, was activated at both time points upon PPARγ deletion, which is known to be suppressed during MASLD progression (Figure 3, E and F). Gene Ontology, Kyoto Encyclopedia of Genes and Genomes, and Reactome analyses further showed significant enrichment for pathways governing lipid and cholesterol metabolism, fatty acid degradation, peroxisome function, PPAR signaling, inflammatory response, extracellular matrix organization, and glucose homeostasis in PPARγ-KO mice (Figure 3, G and H and Supplemental Tables S3 and S4).

**Figure 4:**
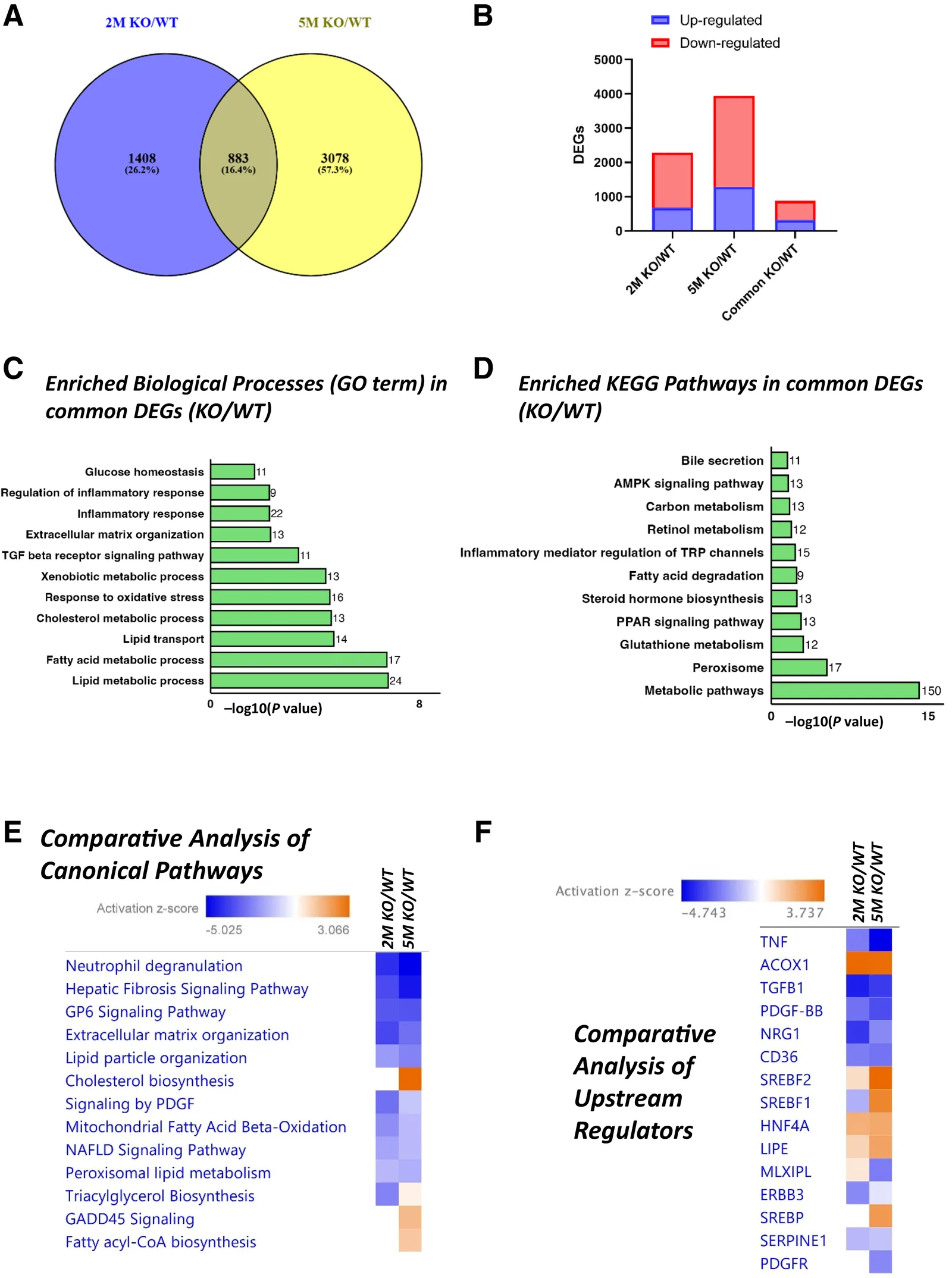
Comparative transcriptomics analysis at 2 months (2M) and 5 months (5M) reveals a time-dependent metabolic reprogramming in hepatocyte-specific peroxisome proliferator-activated receptor (PPAR) gamma-knockout (KO) mice. **A:** Venn diagrams showing overlap of differentially expressed genes (DEGs) at 2M and 5M. **B:** Bar graphs showing up and down-regulated DEGs [KO/wild-type (WT)] at 2M and 5M, and in common. **C** and **D:** Enriched Gene Ontology (GO) biological processes (**C**) and enriched Kyoto Encyclopedia of Genes and Genomes (KEGG) pathways (**D**) in common DEGs at 2M and 5M. **E** and **F:** Comparative canonical pathway (**E**) and comparative upstream regulator analysis (**F**) of DEGs (KO vs WT) from 2M and 5M, analyzed by using Ingenuity Pathway Analysis. *n* = 3.

Further analysis of transcriptomic data revealed that only a small fraction (16%; 883 genes) of DEGs were common at both 2 and 5 months in PPARγ-KO versus wild-type livers, highlighting the temporal shift in dynamics of regulation by PPARγ (Figure 4, A and B). However, DAVID analysis of these common DEGs still showed significant enrichment of key pathways involved in lipid metabolism/transport, inflammatory response, extracellular matrix organization, and glucose homeostasis (Figure 4, C and D and Supplemental Table S5), highlighting the central role of hepatocyte PPARγ in orchestrating MASLD pathogenesis. Lastly, a direct comparison of canonical pathways and upstream regulators altered at 2 and 5 months was performed by using IPA, which highlighted the temporal reprogramming of MASLD upon PPARγ deletion. Comparative canonical pathway analysis confirmed sustained inhibition of hepatic fibrosis, inflammation, lipid particle organization, and nonalcoholic fatty liver disease progression at both 2 and 5 months; selective activation of cholesterol and fatty acid biosynthesis emerged only at 5 months, reflecting adaptive metabolic responses (Figure 4E). Comparative upstream regulator analysis showed that key inflammatory and fibrotic mediators (tumor necrosis factor and TGF-β1) remained strongly inhibited at both 2 and 5 months, and strong SREBP activation emerged selectively at 5 months (Figure 4F). Interestingly, both comparative canonical pathway and upstream regulator analysis revealed consistent inhibition of platelet-derived growth factor signaling at 2 and 5 months, which is an important signal from hepatocytes to stellate cells driving their activation (Figure 4, E and F).

Collectively, these findings show that hepatocyte-specific PPARγ ablation drives extensive hepatic gene expression reprogramming, characterized by robust alteration of metabolic and lipid regulatory networks and marked suppression of inflammatory and fibrotic signaling.

### Temporal Modulation of Fatty Acid and Triglyceride Synthesis Enzyme/Gene Expression by Hepatocyte PPARγ Ablation

**Figure 5:**
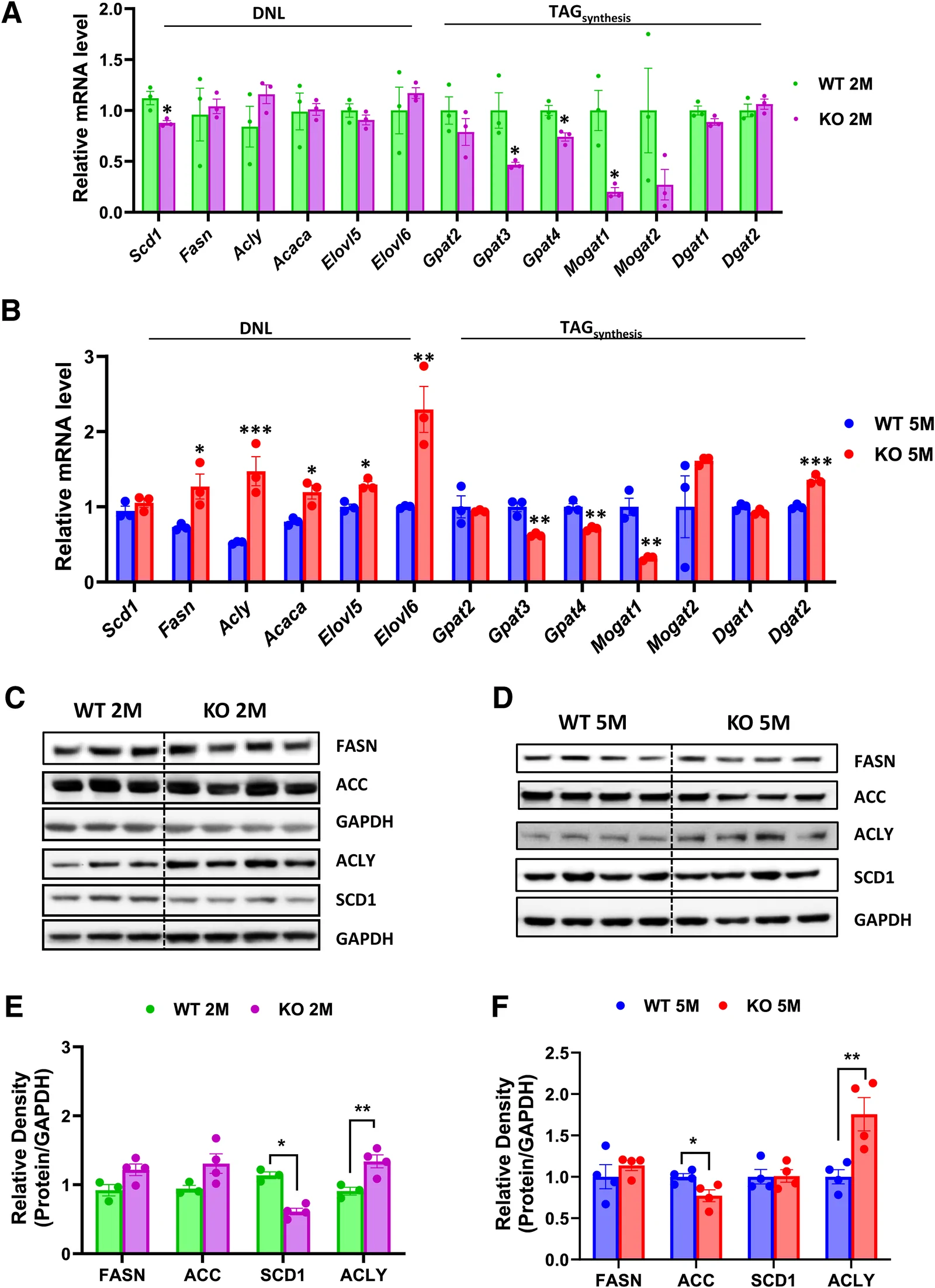
Temporal modulation of fatty acid and triglyceride synthesis enzyme/gene expression by hepatocyte peroxisome proliferator-activated receptor gamma ablation. **A–F:** mRNA expression of *de novo* lipogenesis (DNL) and triacylglycerol (TAG) synthesis genes (**A** and **B**), Western blot analysis of key DNL enzymes (ACC, FASN, SCD1, and ACLY) (**C** and **D**), and quantification of their protein expression normalized to glyceraldehyde-3-phosphate dehydrogenase (GAPDH) (**E** and **F**) in wild-type (WT) and knockout (KO) livers at 2 months (2M) and 5 months (5M). *n* = 3/4. ∗*P* < 0.05, ∗∗*P* < 0.01, ∗∗∗*P* < 0.001.

The profound remodeling of lipid metabolic and regulatory pathways observed after hepatocyte-specific PPARγ deletion prompted a detailed investigation into its impact on key lipogenic gene and protein expression. To elucidate the molecular mechanisms underlying the attenuated hepatic steatosis in PPARγ-KO mice, the expression of critical fatty acid synthesis enzymes, including acetyl-CoA carboxylase (ACC), ATP citrate lyase (ACLY), fatty acid synthase (FASN), and stearoyl-CoA desaturase 1 (SCD1), were analyzed in liver tissues from wild-type and PPARγ-KO mice after 2 and 5 months of feeding FFD. Hepatocyte-specific PPARγ ablation modulated lipogenic gene and protein expression in a time-dependent manner. Notably, *Scd1* expression was significantly down-regulated in PPARγ-KO mice at both the mRNA and protein level after 2 months but not at 5 months (Figure 5, A–F).

Interestingly, mRNA levels of DNL genes, including fatty acid synthesis genes such as *Fasn*, *Acly*, and *Acaca* (encodes for ACC) and fatty acid elongases such as *Elovl5* and *Elovl6*, were up-regulated at 5 months, consistent with a shift to activation of fatty acid biosynthesis and SREBF1 signaling observed in pathway analysis at the transcriptional level (Figure 5B). However, increased mRNA expression of fatty acid synthesis genes was not reflected in the protein analyses, except for ACLY, which displayed increased levels at both time points (Figure 5, C–F). ACC protein expression was significantly reduced at 5 months in PPARγ-KO mice, even though its mRNA expression was higher (Figure 5, B, D, and F). Lastly, several of the genes involved in esterification of fatty acids, by adding glycerol moieties (ie, triacylglycerol synthesis), including *Mogat1, Gpat3,* and *Gpat4,* were consistently down-regulated at both 2 and 5 months in PPARγ-KO mice (Figure 5, A and B).

Together, these findings suggest only a modest effect of hepatocyte-specific PPARγ ablation on impairing DNL and reveal compensatory regulatory mechanisms at the transcriptional level that emerge in the absence of PPARγ in an attempt to enhance DNL during a prolonged dietary challenge.

### Hepatocyte-Specific PPARγ Deletion Induces Lipolysis and Suppresses Lipid Uptake/Storage Genes/Enzymes

**Figure 6:**
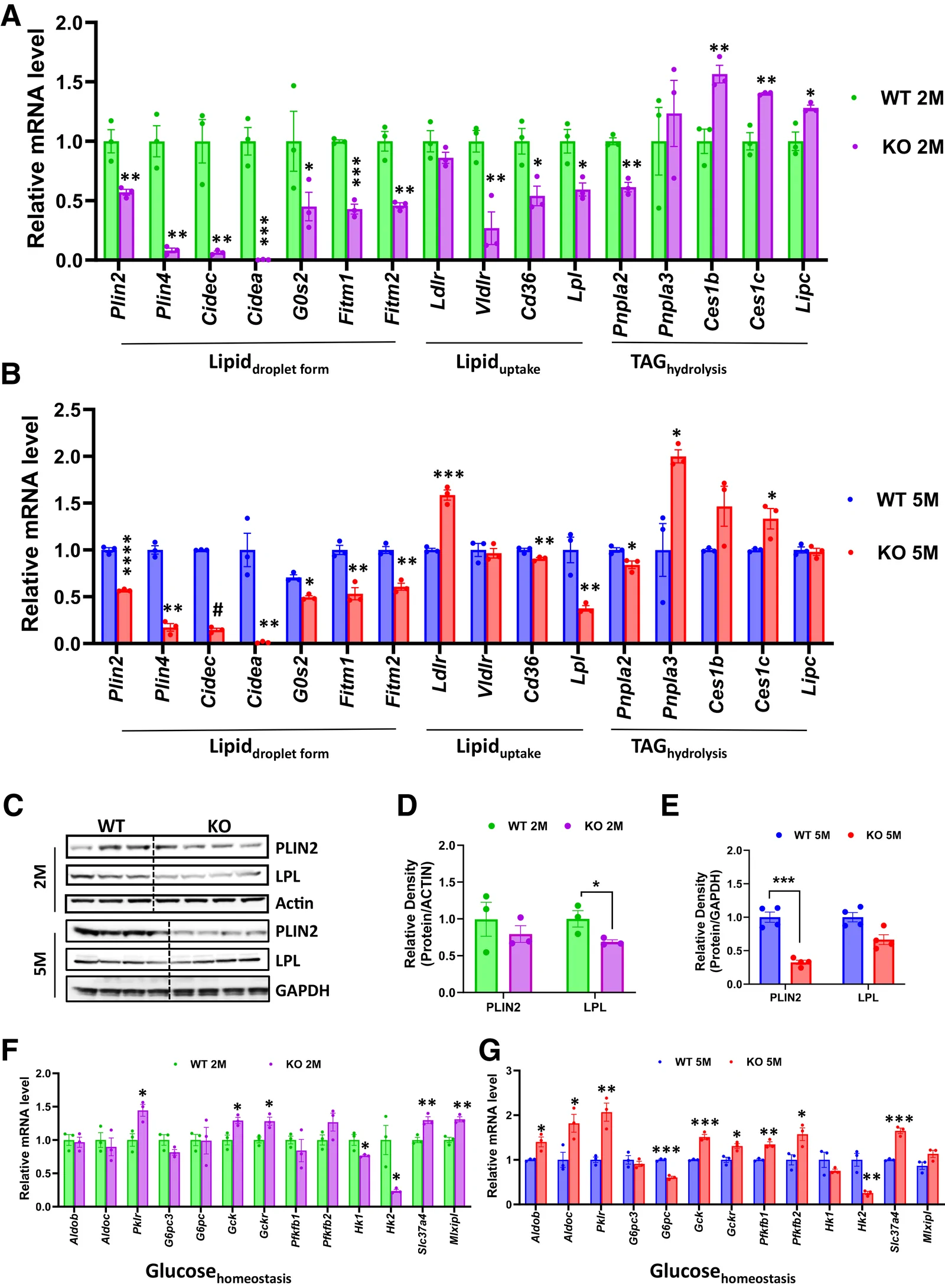
Altered expression of lipid and glucose metabolism genes/proteins in hepatocyte-specific peroxisome proliferator-activated receptor gamma**-**knockout (KO) mice. **A–G:** mRNA expression of lipid droplet formation/stabilization, lipid uptake, and TAG hydrolysis genes (**A** and **B**), Western blot analysis of perilipin 2 and lipoprotein lipase (LPL) (**C**), quantification of their protein expression (**D** and **E**), and mRNA expression of glucose metabolism genes (**F** and **G**) in wild-type (WT) and KO livers at 2 months (2M) and 5 months (5M). *n* = 3/4. ∗*P* < 0.05, ∗∗*P* < 0.01, ∗∗∗*P* < 0.001, ∗∗∗∗*P* < 0.0001, ^#^*P* < 0.00001. GAPDH, glyceraldehyde-3-phosphate dehydrogenase.

In PPARγ-KO livers, key genes involved in lipid droplet formation/stabilization and blocking lipolysis, including perilipins (*Plin2* and *Plin4),* fat storage–inducing transmembrane proteins *(Fitm1* and *Fitm2), G0s2, Cidea,* and *Cidec* (also known as fat-specific protein 27), were significantly down-regulated at both 2 and 5 months (Figure 6, A and B). In contrast, several of the lipolytic genes, including *Ces1c, Ces1b, Lipc*, and *Pnpla3*, were up-regulated at 2 and/or 5 months; the exception was *Pnpla2*, which was down-regulated in PPARγ-KO mice (Figure 6, A and B). Furthermore, expression of genes regulating fatty acid/lipoprotein uptake and transport (including *Cd36, Vldlr*, and *Lpl)* were mostly down-regulated at 2 and/or 5 months, except *Ldlr*, which was induced at 5 months (Figure 6, A and B).

Protein analyses confirmed reduced expression of a major perilipin, PLIN2, at 5 months in PPARγ-KO mice (Figure 6, C–E). Further protein expression of lipoprotein lipase, which regulates hydrolysis of triglycerides in lipoproteins to free fatty acids for uptake by hepatocytes, was reduced at 2 months in PPARγ-KO mice (Figure 6, C–E). In addition to alteration of lipid metabolism genes, several of the important genes involved in regulating glucose homeostasis were significantly altered in PPARγ-KO mice (Figure 6, F and G). Overall, transcriptomic profiling further revealed broad remodeling of hepatic lipid and glucose metabolism genes, underscoring the extensive metabolic reprogramming induced by hepatocyte-specific PPARγ ablation (Supplemental Figure S4).

### Targeted Loss of Hepatocyte PPARγ Reduces Fibrogenic Signaling in MASLD

**Figure 7:**
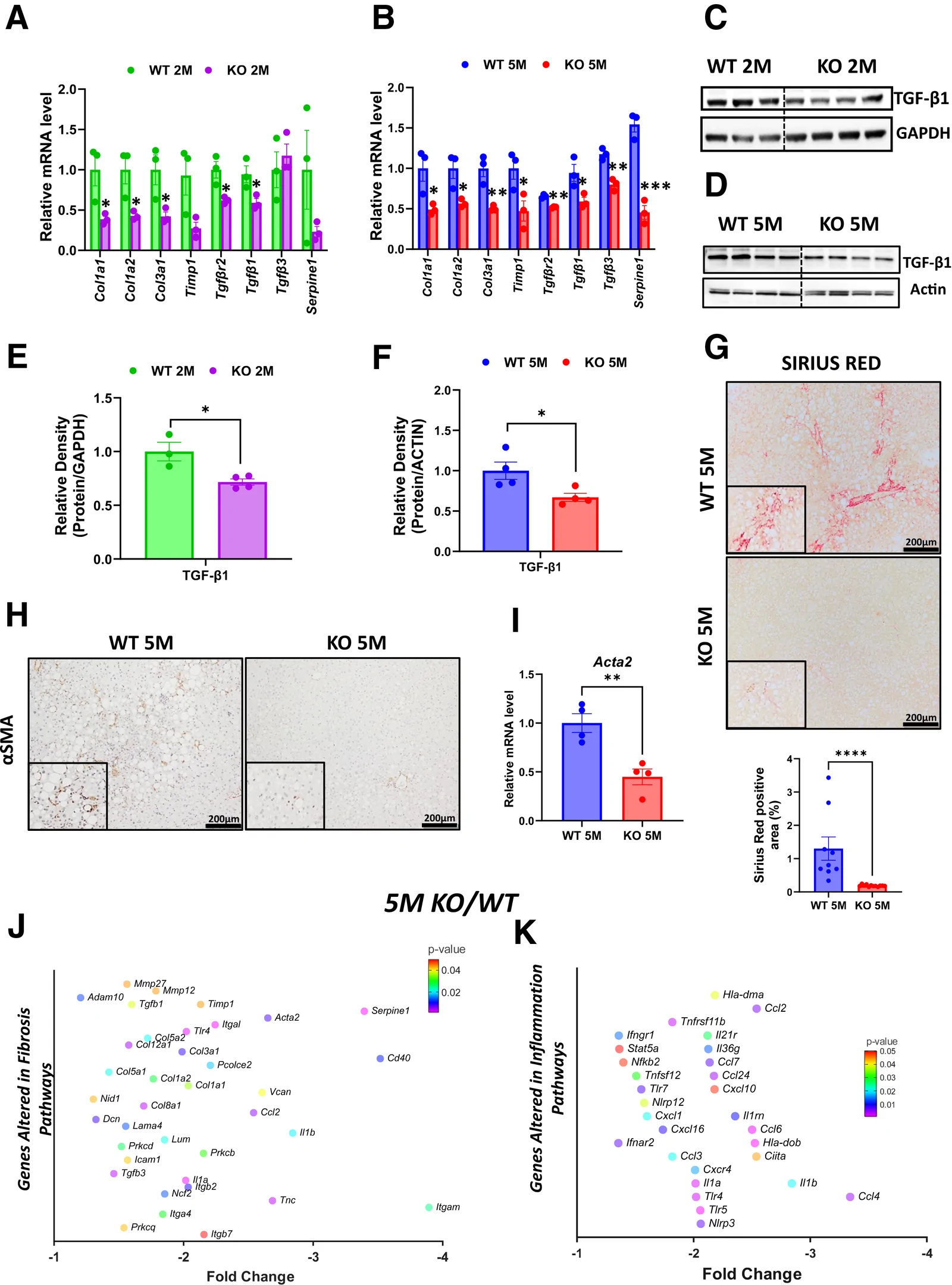
Hepatocyte-specific peroxisome proliferator-activated receptor gamma (PPARγ) deletion attenuates hepatic fibrosis signaling. **A–F:** mRNA expression of fibrogenic genes (**A** and **B**), Western blot analysis of transforming growth factor-β1 (TGF-β1) (**C** and **D**), and its densitometric analysis at 2 months (2M) and 5 months (5M) in knockout (KO) and wild-type (WT) livers (**E** and **F**). **G** and **H:** Sirius Red staining (along with its quantification) (**G**) and α-smooth muscle actin (α-SMA) staining of liver sections from KO and WT mice at 5 months (**H**). **I:** Bar graphs show mRNA expression of *Acta2* gene (encodes α-SMA) at 5 months in KO and WT livers. **J** and **K:** Bubble plot illustrates fold changes and *P* values of differentially expressed hepatic fibrosis and extracellular matrix organization genes (**J**) and inflammatory pathway genes (**K**) in KO versus WT livers at the 5M time point. Bubble color intensity indicates *P* value of significance. *n* = 3/4. ∗*P* < 0.05, ∗∗*P* < 0.01, ∗∗∗*P* < 0.001, ∗∗∗∗*P* < 0.0001. Scale bar = 200 μm (**G** and **H**). GAPDH, glyceraldehyde-3-phosphate dehydrogenase.

Hepatocyte-specific deletion of PPARγ profoundly modulated the fibrogenic response during MASLD progression, in addition to reprogramming hepatic lipid metabolism. PPARγ-KO livers displayed significantly reduced mRNA levels of key collagen genes (*Col1a1, Col1a2,* and *Col3a1*), the tissue inhibitor of metalloproteinases *Timp1*, and extracellular matrix modulator *Serpine1* at both 2 and 5 months compared with controls (Figure 7, A and B). Expression of *Tgfb1,* a central mediator of hepatic fibrogenesis, and its receptor *Tgfbr2* were also markedly decreased in PPARγ-KO mice at both time points (Figure 7, A and B). Transcriptomic profiling of PPARγ-KO livers further revealed broad down-regulation of extracellular matrix remodeling genes, including several other collagens, matrix regulators, and extracellular matrix components (Figure 7J and Supplemental Figure S5A). Some of these transcriptional changes were confirmed at the protein level by immunoblotting, which revealed significantly lower TGF-β1 abundance in PPARγ-KO livers at both 2 and 5 months (Figure 7, C–F).

Consistent with these findings, IPA predicted down-regulation of the TGF-β1 signaling network (Figure 3, E and F and Supplemental Table S6). Sirius Red staining and its quantification revealed minimal collagen deposition in PPARγ-KO mice, contrasting with observable fibrosis in control mice (Figure 7G). Furthermore, α-smooth muscle actin/Acta2 expression, a marker of activated hepatic stellate cells, was significantly reduced in PPARγ-KO livers (Figure 7, H and I). It is important to note that these findings reflect the consequences of hepatocyte-specific PPARγ deletion, and the effects are specific to PPARγ in hepatocytes. By contrast, systemic PPARγ activation reportedly reduces fibrosis by suppressing hepatic stellate cell activation, whereas systemic PPARγ inhibition or deletion can exacerbate fibrogenesis.^30^^,^^31^ Thus, the role of PPARγ in liver fibrosis is cell type dependent and differs between hepatocyte-targeted and systemic modulation. In addition to reduced fibrogenic signaling in PPARγ-KO mice, inflammatory signaling genes were suppressed in these mice, with lower expression of several chemokines and cytokines, adhesion molecules, and integrins, consistent with earlier pathway analysis showing inhibition of key inflammatory upstream regulators (Figure 7K and Supplemental Figure S5B). Overall, these findings highlight a coordinated reduction in inflammation, matrix remodeling, and fibrogenic signaling in the absence of hepatocyte PPARγ during the progression of hepatic fibrosis in MASLD.

### PPARγ Regulates HNF4α during MASLD

**Figure 8:**
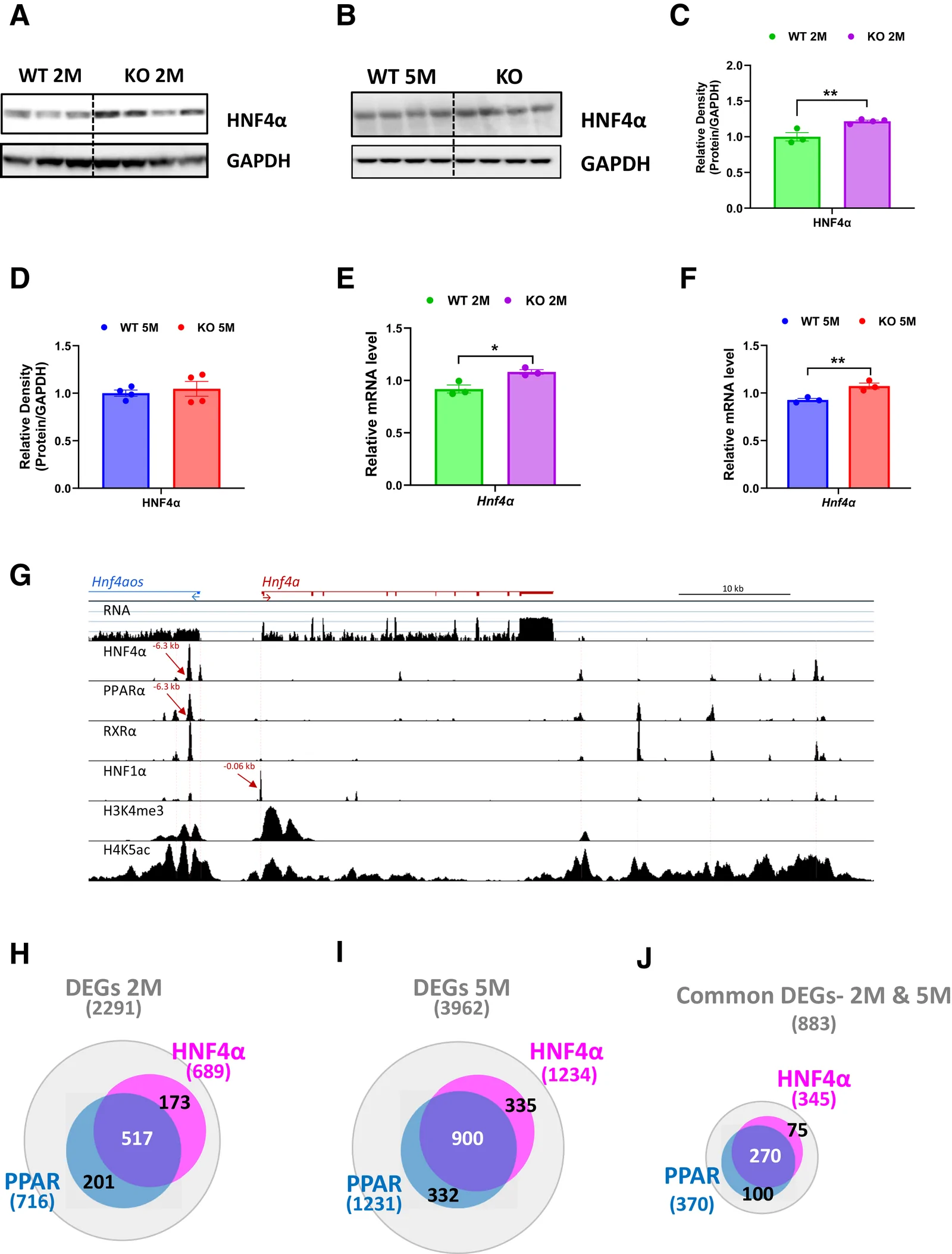
Hepatocyte-specific peroxisome proliferator-activated receptor gamma (PPARγ) ablation increases hepatocyte nuclear factor 4α (HNF4α) expression in metabolic dysfunction–associated steatotic liver disease. **A–F:** Western blot analysis of HNF4α (**A** and **B**), quantification of HNF4α protein levels normalized to glyceraldehyde-3-phosphate dehydrogenase (GAPDH) (**C** and **D**), and mRNA expression of HNF4α (**E** and **F**) at 2 months (2M) and 5 months (5M) in knockout (KO) and wild-type (WT) livers. **G:** Analysis of published chromatin immunoprecipitation (ChIP) sequencing data sets of normal liver showing co-regulation of *Hnf4a* by PPAR and HNF4α. From the top, the plot shows RNA-sequencing characterization of transcripts, and ChIP-sequencing analysis of HNF4α, PPAR alpha (PPARα) (a surrogate for PPARγ), its heterodimeric partner retinoid X receptor alpha (RXRα), hepatocyte nuclear factor 1α (HNF1α), and chromatin modifications H3K4me3 and H4K5ac (transcriptionally active regions). Individual enhancers align with depressions in the H4K5ac-positive regions, where chromatin is depleted (**red lines**). Note that several enhancers bind both HNF4α and PPARα. HNF1α binds near the promoter, a region that does not bind HNF4α or PPARα. The **red arrows** mark the regions used for the ChIP studies of PPARγ and HNF4α (Supplemental Figure S8, A and B). **H–J:** Association of PPAR and HNF4α binding (from ChIP sequencing) with differentially expressed genes (DEGs) (from RNA sequencing) in PPARγ-KO mice: Overlap of DEGs in PPARγ-KO mice with HNF4α, and PPAR peak sets at 2 months (2M) (**H**), 5 months (5M) (**I**), and both (**J**). *n* = 3/4. ∗*P* < 0.05, ∗∗*P* < 0.01.

Given the extensive metabolic and fibrogenic reprogramming observed upon hepatocyte-specific PPARγ deletion, it was sought to identify master transcription factors underlying the protection against MASLD progression. HNF4α, a key regulator of hepatocyte identity and metabolism, emerged as a prime candidate considering it was among the top upstream regulators activated consistently at both 2 and 5 months in PPARγ-KO mice according to the IPA (Figure 3, E and F). The HNF4α downstream gene network remained extensively activated at both time points in PPARγ-KO mice (Supplemental Figures S6 and S7 and Supplemental Table S7). Increased HNF4α activity was accompanied by its elevated protein expression in PPARγ-KO mice at 2 months but not at 5 months (Figure 8, A–D). Interestingly, mRNA levels of HNF4α were significantly up-regulated consistently at both 2 and 5 months in PPARγ-KO mice, indicating that PPARγ represses HNF4α at the transcriptional level (Figure 8, E and F). This is an important observation given the limited understanding of HNF4α transcriptional regulation.

Analysis of previously published ChIP-seq data sets revealed PPAR and retinoid X receptor co-occupancy within *Hnf4a* regulatory regions,^32^ alongside HNF4α autoregulatory binding and active histone marks. This indicates a direct co-regulation of *Hnf4a* by PPAR and HNF4α, with positional analysis indicating frequent binding to the same enhancers (Figure 8G). Due to unavailability of a ChIP-seq data set of PPARγ in mouse liver, PPARα was used as a surrogate marker. PPARα and PPARγ have virtually identical DNA binding,^27^ and the strength and position of their binding sites are highly concordant in mouse liver.^20^ The strongest PPARα and HNF4α-binding enhancer site (Figure 8G) within the *Hnf4a* regulatory region was confirmed by using PPARγ ChIP analysis. The HNF1α binding site near the *Hnf4a* promoter (–0.06 kb), a region that does not bind HNF4α or PPARα, served as a negative control (Figure 8G). ChIP validated direct PPARγ binding to the PPARα and HNF4α-binding enhancer at –6.3 kb under FFD conditions but not to the HNF1α-binding promoter region at –0.06 kb (Figure 8G and Supplemental Figure S8A). Previously established HNF4α binding at the enhancer at –6.3 kb was also confirmed under FFD conditions with HNF4α ChIP (Figure 8G and Supplemental Figure S8B).

Together with integrative functional genomics showing co-occupancy on active chromatin marked by H3K4me3 and H4K5ac, these data establish a cohesive mechanistic model in which PPARγ and HNF4α together directly regulate *Hnf4a* expression during MASLD. These findings are consistent with previously published evidence (ChIP studies) showing occupancy of both PPARγ and HNF4α at multiple shared genomic loci.^20^ Both transcription factors bind the same regulatory regions of lipid metabolism genes, *Scd1*, *Fasn*, and *Plin2*, with PPARγ occupancy enriched more under steatotic (FFD) conditions, whereas HNF4α binding was enhanced more under non-steatotic conditions.^20^

### PPARγ Co-regulation with HNF4α to Drive Metabolic Reprogramming in MASLD

To further explore HNF4α and PPAR interactions at a global level on all genes differentially regulated by PPARγ in this study, published ChIP-seq peak libraries for PPAR and HNF4α, within active chromatin regions, were correlated with DEGs in PPARγ-KO mice at both 2 and 5 months (Figure 8, H–J). Among DEGs in PPARγ-KO mice, PPAR and HNF4α binding was observed in 31.2% and 30.1% of genes at 2 months and 31.1% and 31.2% at 5 months, respectively (Figure 8, H and I). Within the common DEGs between 2 and 5 months, 41.9% and 39.1% were bound by PPAR and HNF4α (Figure 8J). Among DEGs with direct PPAR binding sites, HNF4α binding was also observed at 72.1%, 73.0%, and 72.9% of these genes at 2 months, 5 months, and both 2 and 5 months (Figure 8, H–J); this finding indicates that HNF4α might be an important contributor to PPARγ-driven transcriptomics reprogramming during MASLD. Furthermore, >55% of these genes consistently showed overlapping HNF4α and PPAR binding sites, indicating significant co-regulation.

Canonical pathway analysis of these common HNF4α-PPAR target DEGs (Supplemental Table S8) revealed enrichment in lipid metabolism, glucose homeostasis, and inflammatory response pathways (Supplemental Figure S9); representative co-bound DEGs are shown in Supplemental Figure S10, including *Plin2*, *Bhmt*, *Acox1*, and *Serpine1*. Of these, *Plin2* is of special interest, as results showed that Plin2 mRNA and protein levels were both decreased in PPARγ-KO mice, consistent with reduced lipid droplet formation and hepatic steatosis. Our earlier ChIP study also confirmed binding of both PPARγ and HNF4α to the same enhancer site of *Plin2* under FFD conditions.^20^

Another DEG in the current study, *Bhmt,* which has several common HNF4α and PPAR binding sites in the regulatory region (Supplemental Figure S10), was consistently reported to be altered in PPARγ-KO mice in a previous study and was recognized as a critical contributor to protection against the MASLD phenotype in these mice.^19^ *Bhmt* encodes betaine-homocysteine S-methyltransferase, an important enzyme involved in methionine metabolism and known to negatively regulate lipotoxic events. Overall, this analysis indicates that PPARγ co-regulation with HNF4α might be an important driver of metabolic reprogramming in MASLD.

## Discussion

The current study advances the understanding of hepatocyte-specific PPARγ function in MASLD by integrating longitudinal transcriptomic profiling with a physiologically relevant FFD model.^20^^,^^21^ First, the study showed the clinical relevance of PPARγ activation in the liver (specifically in hepatocytes) during MASLD. PPARγ expression and activity were both increased in hepatocytes of patients with MASH based on analysis of a published single-nuclei RNA-sequencing data set. Consistent with human liver tissue from patients with MASH, significant up-regulation and activation of the PPARγ downstream gene network were observed in livers of mice subjected to FFD for 2 and 5 months. This conserved activation across species underscores the translational value of the FFD model for studying the role of PPARγ in MASLD pathogenesis and supports the hypothesis that hepatocyte PPARγ might be a key driver of metabolic and fibrotic changes in fatty liver disease.^19^

A major strength of the current study lies in its longitudinal design, with analyses performed at both 2 and 5 months of FFD feeding; this approach adds critical temporal resolution to understanding hepatocyte PPARγ function in MASLD at the transcriptomic level. Previous studies have examined both short-term and longer term effects of hepatocyte PPARγ in liver disease,^8^ but many have focused primarily on early time points, which tend to capture initial metabolic changes, including DNL. In the data, Scd1 expression, a key enzyme involved in fatty acid desaturation and an important contributor to lipogenesis, was notably decreased by 2 months, suggesting early suppression of specific lipogenic components. However, by 5 months, the effect on Scd1 was lost, and several of the other key DNL genes were surprisingly induced, indicating a dynamic and stage-dependent regulation of the lipogenic program. Notably, activation of lipolysis-related pathways, and suppression of fatty acid esterification and lipid droplet formation/stabilization pathways, were consistent at both time points, indicating sustained effects on lipid breakdown. These findings highlight the complex temporal regulation of lipid metabolism by PPARγ in MASLD.

In addition to reprogramming lipid metabolism, hepatocyte-specific PPARγ ablation strongly inhibited the downstream transcriptional network of a major fibrosis driver, TGF-β1, along with suppressing its mRNA and protein levels consistently at both 2 and 5 months. Phenotypically, this corresponded with marked reduction in fibrosis (revealed by Sirius Red and α-smooth muscle actin staining), collagen expression, and liver injury markers (alanine aminotransferase and aspartate aminotransferase). These observations align with recent studies reporting that the role of PPARγ in hepatocytes extends beyond simple DNL to include regulation of lipid transport, fatty acid esterification, lipolysis, and fibrogenic pathways.^11^^,^^19^^,^^33^

The current data revealed hepatocyte PPARγ as a pivotal regulator of hepatic lipid accumulation, injury, and fibrogenesis under chronic metabolic stress, while highlighting a novel antagonistic relationship with HNF4α that orchestrates the metabolic reprogramming. Although HNF4α is known to maintain hepatocyte differentiation and metabolic homeostasis, its expression is typically suppressed in MASLD, contributing to hepatocyte dysfunction, steatosis, and fibrosis.^34^, ^35^, ^36^ HNF4α activity, protein expression, and mRNA levels were all up-regulated in PPARγ-KO mice in the murine FFD model. These data also suggest that PPARγ might directly regulate *Hnf4a* transcription through occupancy at its enhancers, as supported by ChIP-seq analysis showing binding of PPAR and HNF4α at the same regulatory regions of *Hnf4a.* ChIP analysis confirmed direct binding of both PPARγ and HNF4α to the same enhancer site in the regulatory region of *Hnf4a* gene in FFD-fed mice liver. The integration of PPAR and HNF4α ChIP-seq data sets with transcriptional profiles from PPARγ-KO mice further indicates that HNF4α co-regulates >70% of genes under direct control of PPARγ during MASLD, underscoring HNF4α as a potential key contributor of the metabolic rewiring that occurs upon loss of hepatocyte PPARγ. Among these shared targets is *Plin2*, a key regulator of lipid droplet formation and lipolysis suppression, which was markedly reduced at both transcript and protein levels in PPARγ-KO mice. Consistent with this finding, the prior ChIP study showed that PPARγ binding to the critical regulatory regions of *Plin2* decreases during MASLD regression in the murine FFD model, concurrently with increased HNF4α binding to the same regulatory regions.^20^ Similarly, binding of PPARγ and HNF4α to the same enhancer sites of critical fatty acid synthesis genes, *Scd1* and *Fasn*, was confirmed in the earlier study in mice fed an FFD,^20^ with an experiment design similar to that of the current study.

Together, these data support a model in which PPARγ and HNF4α co-regulate MASLD gene signature and hepatocyte-specific PPARγ deletion enhances HNF4α activity by its transcription regulation enabling reinstatement of hepatoprotective metabolic programs. This transcriptional switch may represent a key mechanism by which PPARγ loss mitigates steatosis, lipotoxic signaling, and downstream fibrogenic responses. In addition to its central metabolic role, enhanced HNF4α activity likely contributes to reduced fibrosis and inflammation indirectly by maintaining hepatocyte differentiation status and modulating the broader hepatic microenvironment, including stellate cell activation and macrophage responses.^37^

These insights highlight therapeutic opportunities aimed at re-establishing HNF4α-driven transcriptional homeostasis in MASLD. Strategies that selectively augment hepatocyte HNF4α activity, or, conversely, inhibit hepatocyte-specific PPARγ, may offer synergistic benefit by restoring metabolic resilience while limiting secondary inflammatory and fibrogenic pathways. Importantly, given the diverse and cell type–specific functions of PPARγ across the liver and peripheral tissues, therapeutic targeting must be approached with precision to avoid unintended systemic effects. Cell type–selective modulation of PPARγ or targeted enhancement of HNF4α may therefore represent promising avenues for future MASLD interventions.^16^^,^^38^

Limitations of this study include the exclusive use of male mice, which precludes assessment of sex-dependent differences. Furthermore, contribution of specific liver cell types and their intercommunication specifically in relation to observed changes in inflammation and fibrosis signaling pathways require more characterization and need single-cell resolution. Future work should also validate the functional consequences of the PPARγ–HNF4α axis, including direct mechanistic studies of transcriptional repression and autoregulation, and explore translational approaches for hepatocyte-specific modulation.

In conclusion, this study delineates hepatocyte PPARγ as a critical driver of MASLD pathogenesis and identifies its antagonism of HNF4α as one of the key regulatory mechanisms. These findings provide a compelling rationale for cell-specific therapeutic targeting of PPARγ to reprogram hepatic metabolism and mitigate steatosis and fibrosis in MASLD.

## Disclosure Statement

None declared.

## Supplemental Data

Supplemental Figure S1 The visualization of peroxisome proliferator-activated receptor gamma (PPARγ) downstream network activation in human hepatocytes from patients with metabolic dysfunction–associated steatotic liver disease [metabolic dysfunction–associated steatohepatitis (MASH)-associated cirrhosis stage] versus healthy control (HC) individuals (identified by Ingenuity Pathway Analysis of published single-nuclei RNA sequencing data). Positive z-score indicates predicted activation of the pathway.

Supplemental Figure S2 The visualization of peroxisome proliferator-activated receptor gamma (PPARγ) downstream network activation in a mouse fast-food diet (FFD) model. Positive z-score indicates predicted activation of the pathway.

Supplemental Figure S3 SREBF1 downstream gene network inhibition at 2 months (2M) (**A**) and activation at 5 months (5M) (**B**) in knockout (KO) versus wild-type (WT) livers, analyzed by using Ingenuity Pathway Analysis.

Supplemental Figure S4 Bubble plot illustrates fold changes and *P* values of differentially expressed lipid/glucose metabolism genes in knockout (KO) versus wild-type (WT) livers at 2 months (2M) (**A**) and 5 months (5M) (**B**).

Supplemental Figure S5 Bubble plot illustrates fold changes and *P* values of differentially expressed genes associated with fibrosis (**A**) and inflammation (**B**) signaling in knockout (KO) versus wild-type (WT) livers at 2 months (2M).

Supplemental Figure S6 Hepatocyte nuclear factor 4α (HNF4α) downstream gene network activation at 2 months in knockout versus wild-type livers, analyzed by using Ingenuity Pathway Analysis.

Supplemental Figure S7 Hepatocyte nuclear factor 4α (HNF4α) downstream gene network activation at 5 months in knockout versus wild-type livers, analyzed using Ingenuity Pathway Analysis.

Supplemental Figure S8 Peroxisome proliferator-activated receptor gamma (PPARγ) and hepatocyte nuclear factor 4α (HNF4α) chromatin binding in the regulatory region of the *Hnf4**a* gene. Chromatin immunoprecipitation (ChIP) analysis showing PPARγ binding (**A**) and HNF4α binding (**B**) at the –6.3 kb enhancer site within the regulatory region of *Hnf4**a* gene in wild-type mice fed a fast-food diet for 2 months. The promoter-linked hepatocyte nuclear factor 1α site (–0.06 kb), a region that does not bind HNF4α or PPAR, was used as a negative control. These locations are marked with **red arrows** in Figure 8G.

Supplemental Figure S9 Volcano plots showing differentially expressed genes (up-regulated and down-regulated) linked to both peroxisome proliferator-activated receptor (PPAR) and hepatocyte nuclear factor 4α binding sites at 2 months (2M) (**A**) and 5 months (5M) (**B**). Enriched canonical pathways in differentially expressed genes linked to both PPAR and hepatocyte nuclear factor 4α binding sites at 2M (**C**) and 5M (**D**). NAFLD, nonalcoholic fatty liver disease; PDGF, platelet-derived growth factor.

Supplemental Figure S10 Chromatin immunoprecipitation sequencing characterization of peroxisome proliferator-activated receptor alpha (PPARα) and hepatocyte nuclear factor 4α (HNF4α) binding enhancers near *Plin2, Acox1, Bhmt,* and *Serpine1* genes. **A–D:***Plin2* (**A**), *Acox1* (**B**), *Bhmt* (**C**), and *Serpine1* (**D**) all show co-regulation by HNF4α and PPAR. Figure 8G provides a description of these chromatin immunoprecipitation sequencing plots. RXRα, retinoid X receptor alpha.
