# Supplementary material for: Hepatocyte-specific PPARγ Deletion Uncovers Role of an Antagonistic PPARγ–HNF4α Transcriptional Axis in Metabolic Dysfunction–Associated Steatotic Liver Disease Progression
Source: Am J Pathol. 2026 May 12;196(8):1563–80. doi: 10.1016/j.ajpath.2026.04.015 (PMC13494202; doi:10.1016/j.ajpath.2026.04.015)
Supplement: Supplementary file 2 [file mmc10.pdf]

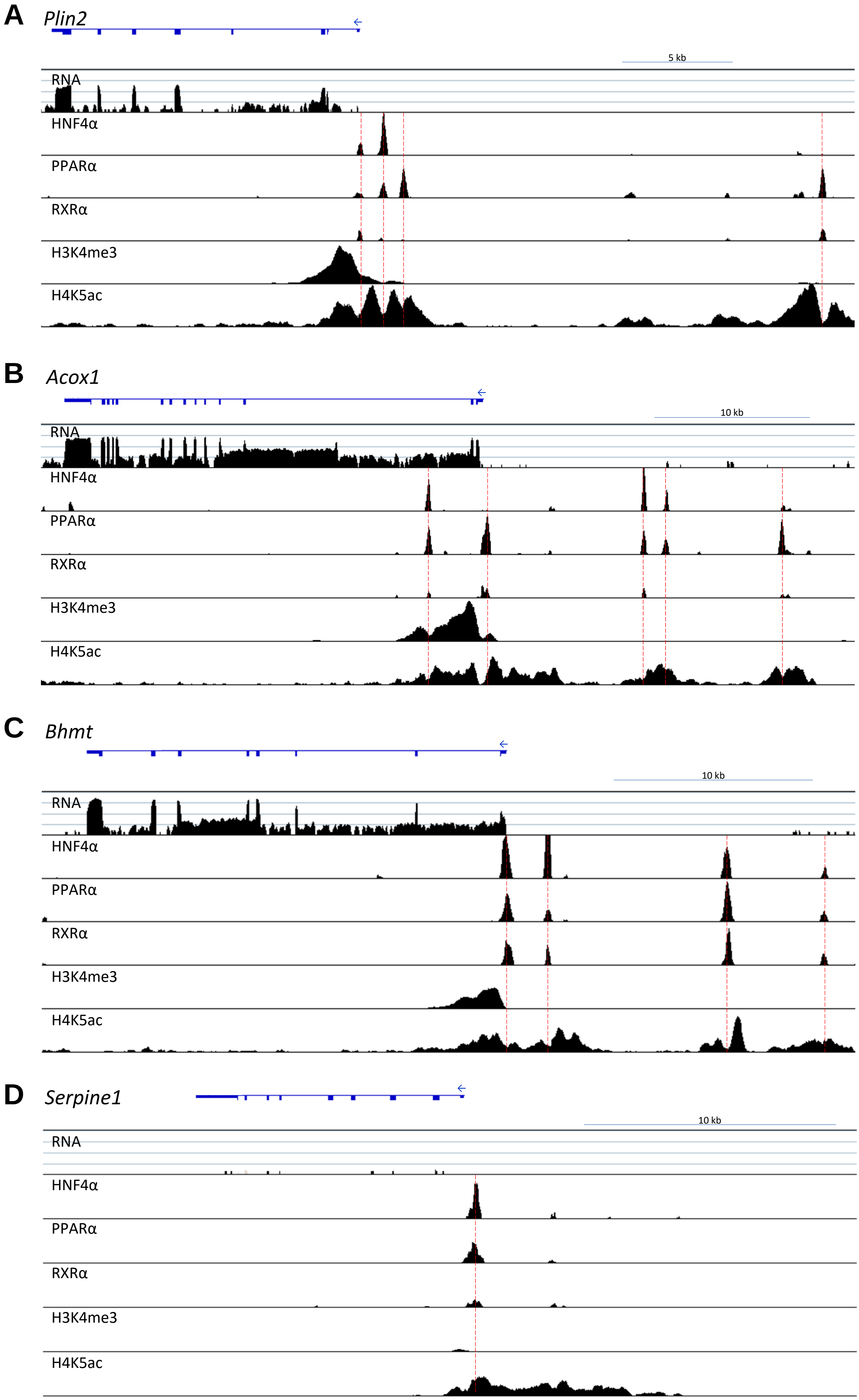
A
Plin2
5 kb
RNA
HNF4α
PPARα
RXRα
H3K4me3
H4K5ac
B
Acox1
10 kb
RNA
HNF4α
PPARα
RXRα
H3K4me3
H4K5ac
C
Bhmt
10 kb
RNA
HNF4α
PPARα
RXRα
H3K4me3
H4K5ac
D
Serpine1
10 kb
RNA
HNF4α
PPARα
RXRα
H3K4me3
H4K5ac
