# Supplementary material for: Hepatocyte-specific PPARγ Deletion Uncovers Role of an Antagonistic PPARγ–HNF4α Transcriptional Axis in Metabolic Dysfunction–Associated Steatotic Liver Disease Progression
Source: Am J Pathol. 2026 May 12;196(8):1563–80. doi: 10.1016/j.ajpath.2026.04.015 (PMC13494202; doi:10.1016/j.ajpath.2026.04.015)
Supplement: Supplementary file 4 [file mmc3.pdf]

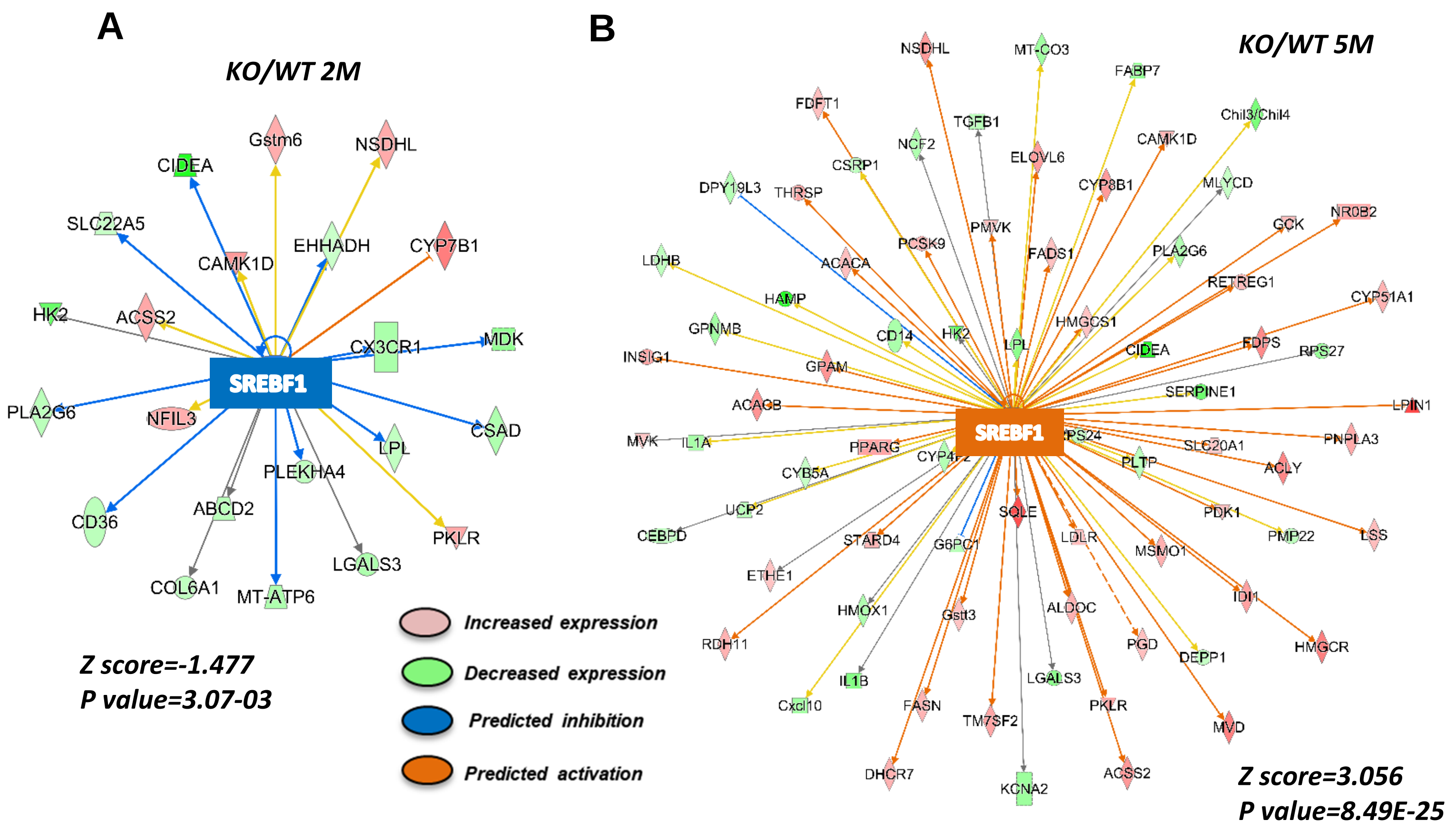

A
KO/WT 2M
SREBF1
Gstm6
NSDHL
CIDEA
SLC22A5
CAMK1D
EHHADH
CYP7B1
HK2
ACSS2
CX3CR1
MDK
PLA2G6
NFIL3
CSAD
LPL
PLEKHA4
CD36
ABCD2
PKLR
LGALS3
COL6A1
MT-ATP6
Z score=-1.477
P value=3.07-03
Increased expression
Decreased expression
Predicted inhibition
Predicted activation
B
KO/WT 5M
SREBF1
NSDHL
MT-CO3
FABP7
FDFT1
TGFB1
NCF2
Chil3/Chil4
CAMK1D
ELOVL6
CSRP1
CYP8B1
MLYCD
DPY19L3
THRSP
NR0B2
PMVK
GCK
PCSK9
FADS1
PLA2G6
LDHB
ACACA
RETREG1
HAMP
CYP51A1
HMGCS1
GPNMB
CD14
HK2
LPL
CIDEA
FDPS
RPS27
INSIG1
GPAM
SERPINE1
ACACB
LPIN1
MVK
IL1A
PPARG
PNPLA3
SLC20A1
ACLY
CYB5A
PLTP
UCP2
SQLE
PDK1
PMP22
LSS
CEBPD
STARD4
G6PC1
LDLR
MSMO1
ETHE1
IDI1
HMOX1
Gstt3
ALDOC
RDH11
PGD
DEPP1
HMGCR
IL1B
LGALS3
Cxcl10
FASN
PKLR
TM7SF2
MVD
DHCR7
ACSS2
KCNA2
Z score=3.056
P value=8.49E-25
