# Supplementary material for: Hepatocyte-specific PPARγ Deletion Uncovers Role of an Antagonistic PPARγ–HNF4α Transcriptional Axis in Metabolic Dysfunction–Associated Steatotic Liver Disease Progression
Source: Am J Pathol. 2026 May 12;196(8):1563–80. doi: 10.1016/j.ajpath.2026.04.015 (PMC13494202; doi:10.1016/j.ajpath.2026.04.015)
Supplement: Supplementary file 5 [file mmc4.pdf]

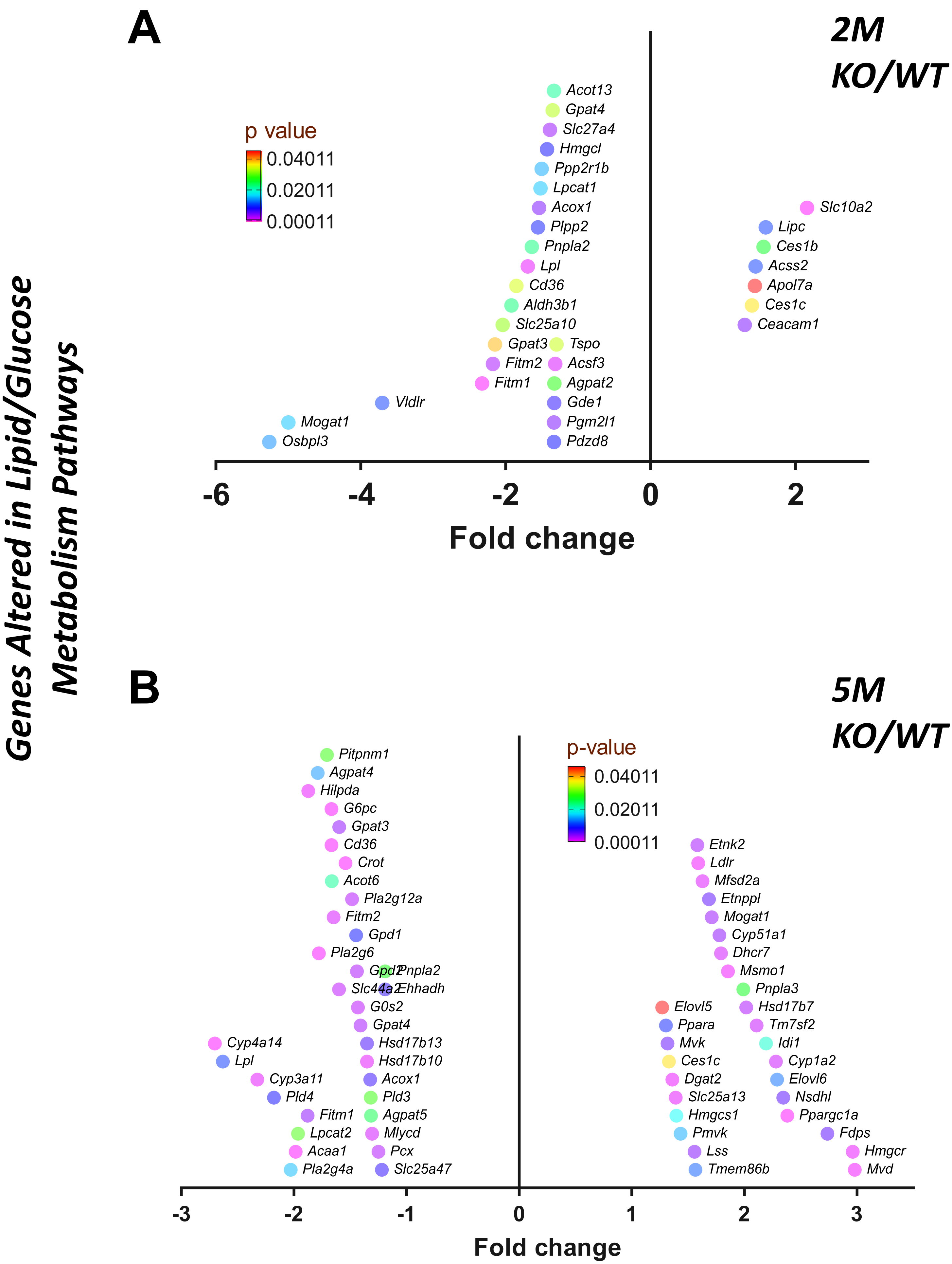
A
2M
KO/WT
p value
0.04011
0.02011
0.00011
Acot13
Gpat4
Slc27a4
Hmgcl
Ppp2r1b
Lpcat1
Acox1
Plpp2
Pnpla2
Lpl
Cd36
Aldh3b1
Slc25a10
Gpat3
Tspo
Fitm2
Acsf3
Fitm1
Agpat2
Vldlr
Gde1
Mogat1
Pgm2l1
Osbpl3
Pdzd8
Slc10a2
Lipc
Ces1b
Acss2
Apol7a
Ces1c
Ceacam1
-6
-4
-2
0
2
Fold change
Genes Altered in Lipid/Glucose Metabolism Pathways
B
5M
KO/WT
p-value
0.04011
0.02011
0.00011
Pitpnm1
Agpat4
Hilpda
G6pc
Gpat3
Cd36
Crot
Acot6
Pla2g12a
Fitm2
Gpd1
Pla2g6
Gpd2
Pnpla2
Slc44a2
Ehhadh
G0s2
Gpat4
Cyp4a14
Hsd17b13
Lpl
Hsd17b10
Cyp3a11
Acox1
Pld4
Pld3
Fitm1
Agpat5
Lpcat2
Mlycd
Acaa1
Pcx
Pla2g4a
Slc25a47
Etnk2
Ldlr
Mfsd2a
Etnppl
Mogat1
Cyp51a1
Dhcr7
Msmo1
Pnpla3
Elovl5
Hsd17b7
Ppara
Tm7sf2
Mvk
Idi1
Ces1c
Cyp1a2
Dgat2
Elovl6
Slc25a13
Nsdhl
Hmgcs1
Ppargc1a
Pmvk
Fdps
Lss
Hmgcr
Tmem86b
Mvd
-3
-2
-1
0
1
2
3
Fold change
