# Supplementary material for: Hepatocyte-specific PPARγ Deletion Uncovers Role of an Antagonistic PPARγ–HNF4α Transcriptional Axis in Metabolic Dysfunction–Associated Steatotic Liver Disease Progression
Source: Am J Pathol. 2026 May 12;196(8):1563–80. doi: 10.1016/j.ajpath.2026.04.015 (PMC13494202; doi:10.1016/j.ajpath.2026.04.015)
Supplement: Supplementary file 6 [file mmc5.pdf]

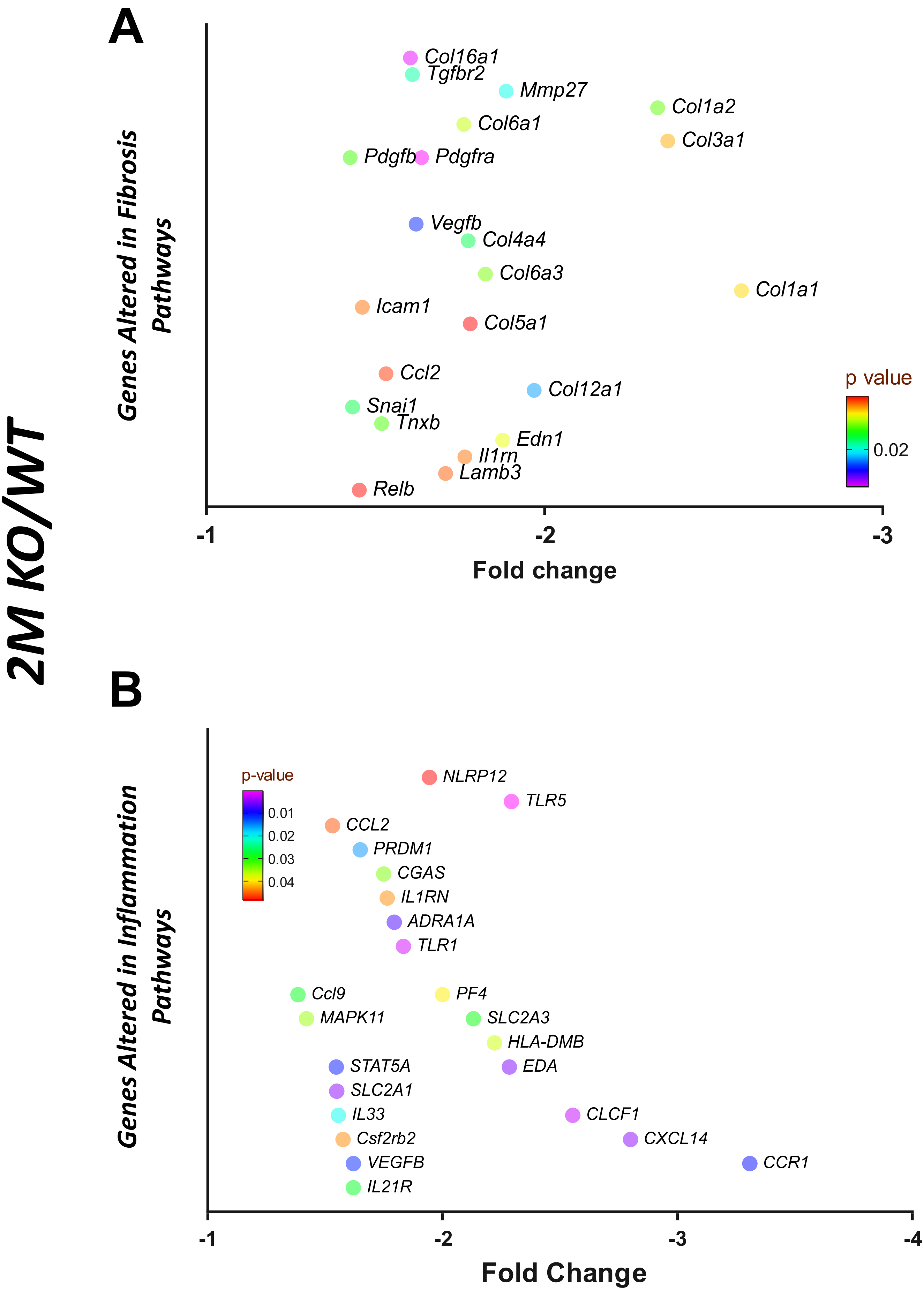

2M KO/WT
A
Genes Altered in Fibrosis Pathways
Col16a1
Tgfbr2
Mmp27
Col1a2
Col6a1
Col3a1
Pdgfb
Pdgfra
Vegfb
Col4a4
Col6a3
Col1a1
Icam1
Col5a1
Ccl2
Col12a1
Snai1
Tnxb
Edn1
Il1rn
Lamb3
Relb
p value
0.02
-1
-2
-3
Fold change
B
Genes Altered in Inflammation Pathways
p-value
0.01
0.02
0.03
0.04
NLRP12
TLR5
CCL2
PRDM1
CGAS
IL1RN
ADRA1A
TLR1
Ccl9
PF4
MAPK11
SLC2A3
HLA-DMB
STAT5A
EDA
SLC2A1
IL33
CLCF1
Csf2rb2
CXCL14
VEGFB
CCR1
IL21R
-1
-2
-3
-4
Fold Change
