# Supplementary material for: Hepatocyte-specific PPARγ Deletion Uncovers Role of an Antagonistic PPARγ–HNF4α Transcriptional Axis in Metabolic Dysfunction–Associated Steatotic Liver Disease Progression
Source: Am J Pathol. 2026 May 12;196(8):1563–80. doi: 10.1016/j.ajpath.2026.04.015 (PMC13494202; doi:10.1016/j.ajpath.2026.04.015)
Supplement: Supplementary file 7 [file mmc6.pdf]

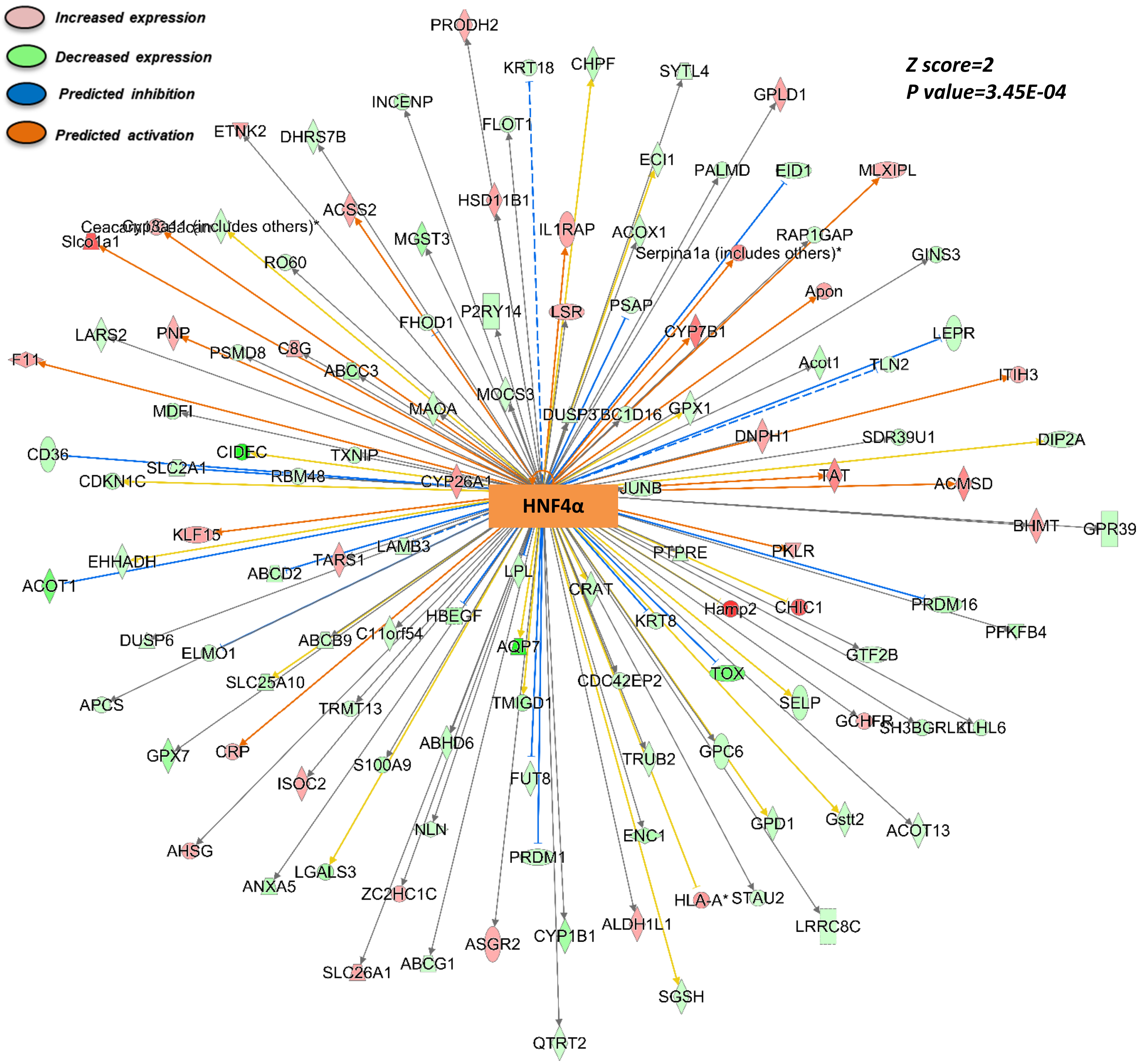

Increased expression
Decreased expression
Predicted inhibition
Predicted activation
Z score=2
P value=3.45E-04
HNF4α
PRODH2
KRT18
CHPF
SYTL4
GPLD1
INCENP
FLOT1
ETNK2
DHRS7B
ECI1
PALMD
EID1
MLXIPL
ACSS2
HSD11B1
IL1RAP
ACOX1
RAP1GAP
Slco1a1
MGST3
Serpina1a (includes others)*
GINS3
RO60
Apon
P2RY14
LSR
PSAP
FHOD1
LARS2
PNP
C8G
CYP7B1
LEPR
F11
PSMD8
ABCC3
Acot1
TLN2
ITIH3
MOCS3
MAOA
GPX1
MDFI
DUSP3
TBC1D16
DNPH1
SDR39U1
DIP2A
CD36
CIDEC
TXNIP
SLC2A1
RBM48
TAT
CDKN1C
CYP26A1
JUNB
ACMSD
BHMT
GPR39
KLF15
LAMB3
PTPRE
PKLR
EHHADH
TARS1
ABCD2
LPL
ACOT1
CRAT
Hamp2
CHIC1
PRDM16
HBEGF
KRT8
PFKFB4
DUSP6
ABCB9
C11orf54
ELMO1
AQP7
GTF2B
TOX
SLC25A10
CDC42EP2
TMIGD1
SELP
APCS
TRMT13
GCHFR
SH3BGRL
KLHL6
GPX7
CRP
ABHD6
TRUB2
GPC6
ISOC2
S100A9
FUT8
AHSG
NLN
ENC1
GPD1
Gstt2
ACOT13
PRDM1
LGALS3
ANXA5
ZC2HC1C
HLA-A*
STAU2
ALDH1L1
LRRC8C
ASGR2
CYP1B1
ABCG1
SLC26A1
SGSH
QTRT2
