# Supplementary material for: Hepatocyte-specific PPARγ Deletion Uncovers Role of an Antagonistic PPARγ–HNF4α Transcriptional Axis in Metabolic Dysfunction–Associated Steatotic Liver Disease Progression
Source: Am J Pathol. 2026 May 12;196(8):1563–80. doi: 10.1016/j.ajpath.2026.04.015 (PMC13494202; doi:10.1016/j.ajpath.2026.04.015)
Supplement: Supplementary file 8 [file mmc7.pdf]

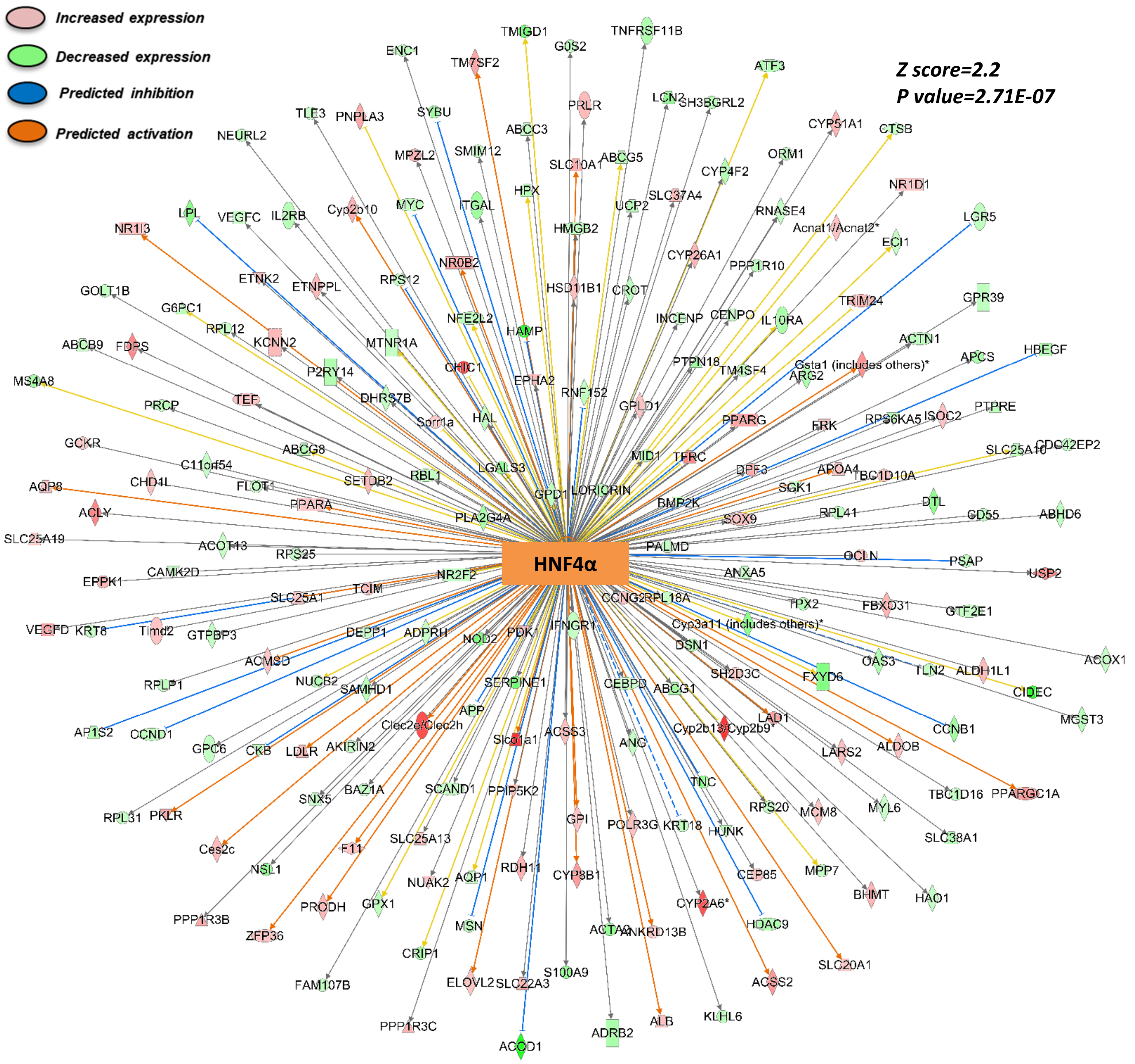
Increased expression
Decreased expression
Predicted inhibition
Predicted activation
Z score=2.2
P value=2.71E-07
HNF4α
TMIGD1
TNFRSF11B
ENC1
G0S2
TM7SF2
ATF3
PRLR
LCN2
SH3BGRL2
TLE3
PNPLA3
SYBU
ABCC3
CYP51A1
CTSB
NEURL2
MPZL2
SMIM12
SLC10A1
ABCG5
ORM1
CYP4F2
HPX
SLC37A4
NR1D1
MYC
ITGAL
UCP2
RNASE4
LPL
VEGFC
IL2RB
Cyp2b10
HMGB2
Acnat1/Acnat2*
LGR5
NR1I3
ECI1
NR0B2
CYP26A1
PPP1R10
ETNK2
ETNPPL
RPS12
HSD11B1
CROT
GOLT1B
TRIM24
GPR39
G6PC1
NFE2L2
INCENP
CENPO
IL10RA
RPL12
HAMP
ACTN1
ABCB9
FDPS
KCNN2
MTNR1A
PTPN18
APCS
HBEGF
P2RY14
CHIC1
TM4SF4
Gsta1 (includes others)*
MS4A8
EPHA2
RNF152
ARG2
DHRS7B
GPLD1
PRCP
TEF
HAL
PPARG
RPS6KA5
ISOC2
PTPRE
Sprr1a
FRK
GCKR
ABCG8
MID1
TFRC
SLC25A10
CDC42EP2
C11orf54
LGALS3
DPF3
APOA4
TBC1D10A
AQP8
CHD1L
SETDB2
RBL1
GPD1
LORICRIN
SGK1
FLOT1
BMP2K
DTL
PPARA
PLA2G4A
SOX9
RPL41
CD55
ABHD6
ACLY
SLC25A19
ACOT13
PALMD
RPS25
OCLN
PSAP
CAMK2D
EPPK1
NR2F2
ANXA5
USP2
TCIM
SLC25A1
CCNG2
RPL18A
TPX2
FBXO31
GTF2E1
VEGFD
KRT8
Timd2
GTPBP3
DEPP1
ADPRH
PDK1
IFNGR1
Cyp3a11 (includes others)*
NOD2
DSN1
ACOX1
ACMSD
OAS3
TLN2
ALDH1L1
SH2D3C
FXYD6
RPLP1
NUCB2
SERPINE1
CEBPD
ABCG1
CIDEC
SAMHD1
MGST3
APP
LAD1
AP1S2
CCND1
Clec2e/Clec2h
ACSS3
Cyp2b13/Cyp2b9*
CCNB1
Slco1a1
ANG
GPC6
CKB
LDLR
AKIRIN2
ALDOB
LARS2
TNC
SCAND1
TBC1D16
PPARGC1A
BAZ1A
PPIP5K2
SNX5
RPS20
MCM8
MYL6
RPL31
PKLR
GPI
POLR3G
KRT18
HUNK
SLC38A1
SLC25A13
Ces2c
F11
NSL1
RDH11
AQP1
NUAK2
CYP8B1
MPP7
CEP85
BHMT
HAO1
PPP1R3B
PRODH
GPX1
CYP2A6*
ZFP36
HDAC9
MSN
ACTA2
ANKRD13B
CRIP1
SLC20A1
S100A9
ACSS2
FAM107B
ELOVL2
SLC22A3
KLHL6
ALB
PPP1R3C
ADRB2
ACOD1
