# Supplementary material for: Hepatocyte-specific PPARγ Deletion Uncovers Role of an Antagonistic PPARγ–HNF4α Transcriptional Axis in Metabolic Dysfunction–Associated Steatotic Liver Disease Progression
Source: Am J Pathol. 2026 May 12;196(8):1563–80. doi: 10.1016/j.ajpath.2026.04.015 (PMC13494202; doi:10.1016/j.ajpath.2026.04.015)
Supplement: Supplementary file 9 [file mmc8.pdf]

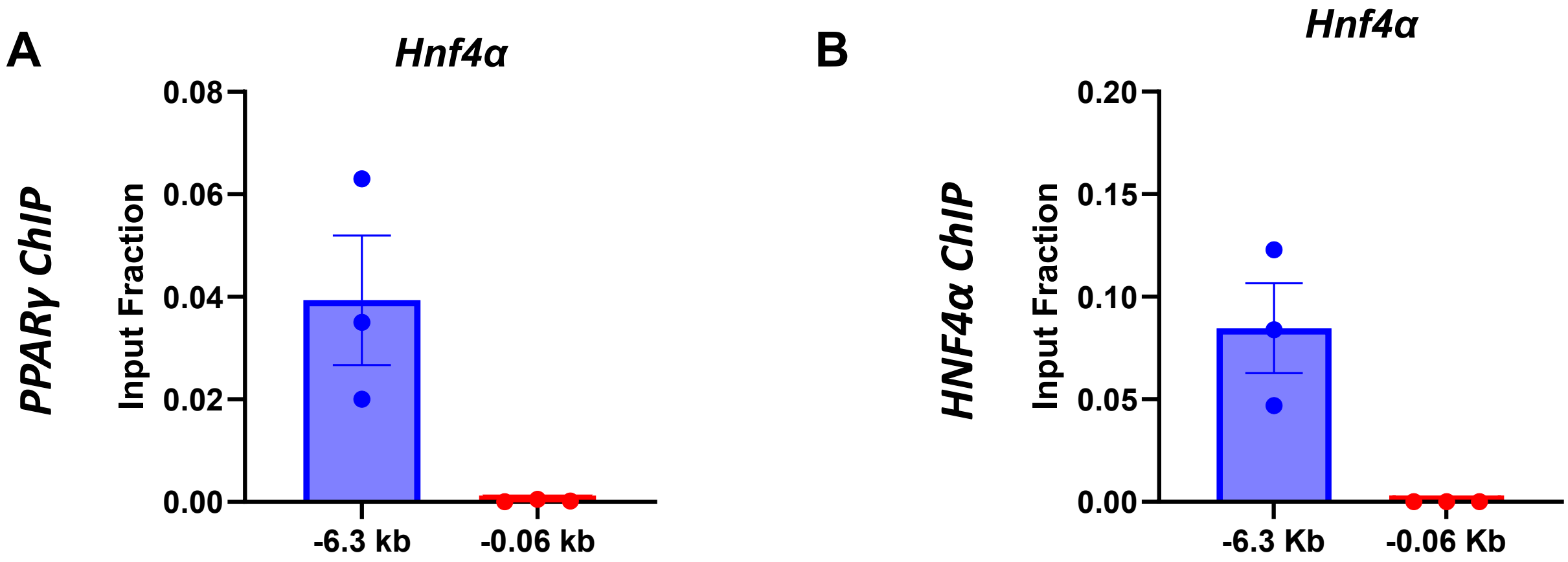

A
Hnf4α
PPARγ ChIP
Input Fraction
0.08
0.06
0.04
0.02
0.00
-6.3 kb
-0.06 kb
B
Hnf4α
HNF4α ChIP
Input Fraction
0.20
0.15
0.10
0.05
0.00
-6.3 Kb
-0.06 Kb
