# Supplementary material for: Hepatocyte-specific PPARγ Deletion Uncovers Role of an Antagonistic PPARγ–HNF4α Transcriptional Axis in Metabolic Dysfunction–Associated Steatotic Liver Disease Progression
Source: Am J Pathol. 2026 May 12;196(8):1563–80. doi: 10.1016/j.ajpath.2026.04.015 (PMC13494202; doi:10.1016/j.ajpath.2026.04.015)
Supplement: Supplementary file 10 [file mmc9.pdf]

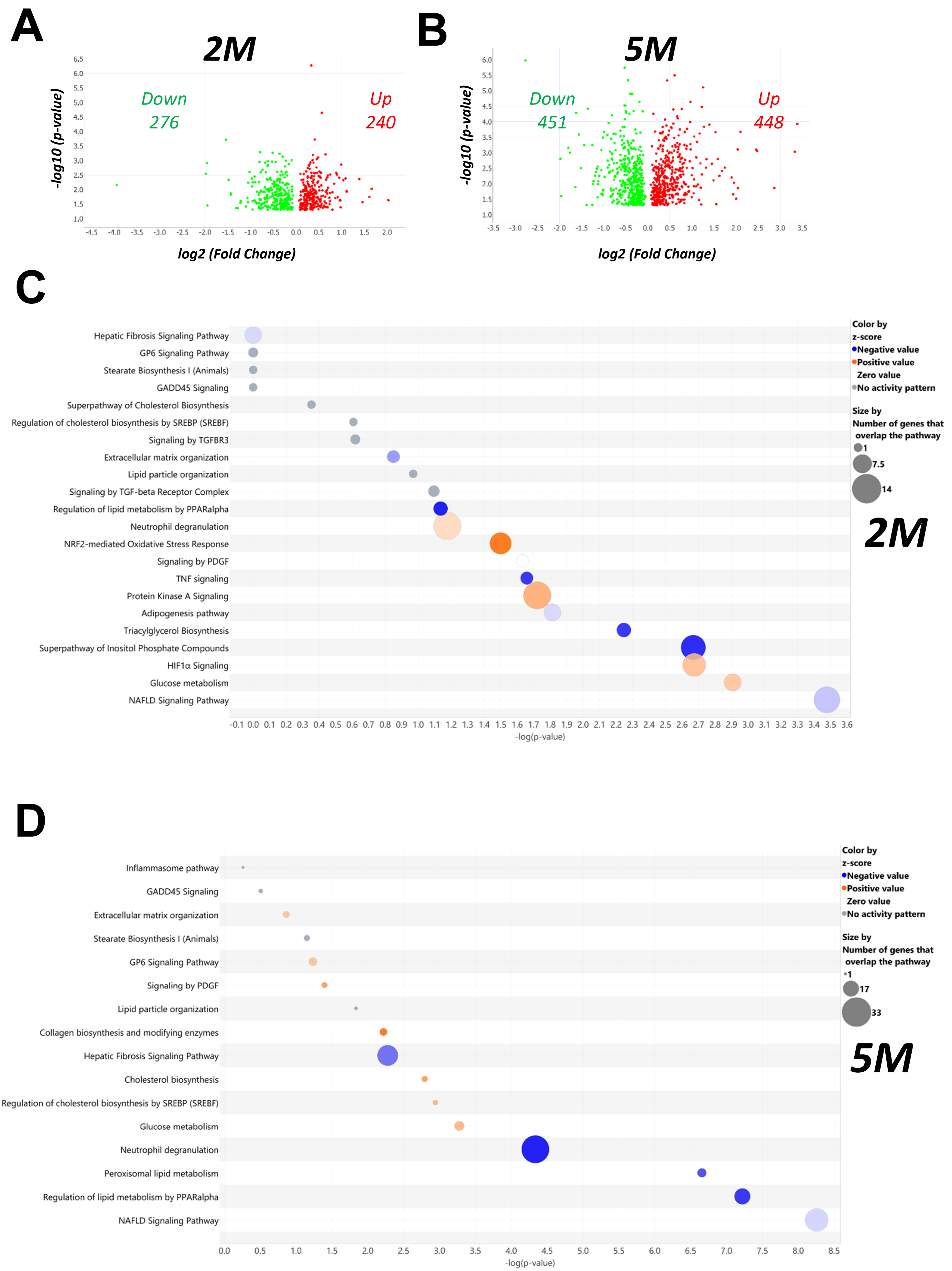

A
2M
Down
276
Up
240
-log10 (p-value)
log2 (Fold Change)
B
5M
Down
451
Up
448
-log10 (p-value)
log2 (Fold Change)
C
Hepatic Fibrosis Signaling Pathway
GP6 Signaling Pathway
Stearate Biosynthesis I (Animals)
GADD45 Signaling
Superpathway of Cholesterol Biosynthesis
Regulation of cholesterol biosynthesis by SREBP (SREBF)
Signaling by TGFBR3
Extracellular matrix organization
Lipid particle organization
Signaling by TGF-beta Receptor Complex
Regulation of lipid metabolism by PPARalpha
Neutrophil degranulation
NRF2-mediated Oxidative Stress Response
Signaling by PDGF
TNF signaling
Protein Kinase A Signaling
Adipogenesis pathway
Triacylglycerol Biosynthesis
Superpathway of Inositol Phosphate Compounds
HIF1α Signaling
Glucose metabolism
NAFLD Signaling Pathway
-log(p-value)
Color by
z-score
Negative value
Positive value
Zero value
No activity pattern
Size by
Number of genes that
overlap the pathway
1
7.5
14
2M
D
Inflammasome pathway
GADD45 Signaling
Extracellular matrix organization
Stearate Biosynthesis I (Animals)
GP6 Signaling Pathway
Signaling by PDGF
Lipid particle organization
Collagen biosynthesis and modifying enzymes
Hepatic Fibrosis Signaling Pathway
Cholesterol biosynthesis
Regulation of cholesterol biosynthesis by SREBP (SREBF)
Glucose metabolism
Neutrophil degranulation
Peroxisomal lipid metabolism
Regulation of lipid metabolism by PPARalpha
NAFLD Signaling Pathway
-log(p-value)
Color by
z-score
Negative value
Positive value
Zero value
No activity pattern
Size by
Number of genes that
overlap the pathway
1
17
33
5M
