# Supplementary material for: Hepatocyte-specific PPARγ Deletion Uncovers Role of an Antagonistic PPARγ–HNF4α Transcriptional Axis in Metabolic Dysfunction–Associated Steatotic Liver Disease Progression
Source: Am J Pathol. 2026 May 12;196(8):1563–80. doi: 10.1016/j.ajpath.2026.04.015 (PMC13494202; doi:10.1016/j.ajpath.2026.04.015)
Supplement: Supplementary file 11 [file mmc11.docx]

**Supplemental Table S1. Top 50 Genes That Were Up-regulated and Down-regulated in**

**PPARγ -KO vs WT Mice on FFD for 2 Months.**

| ***Up genes*** | ***KO/WT*** | ***P value*** | ***Down genes*** | ***KO/WT*** | ***P value*** |
| --- | --- | --- | --- | --- | --- |
| *Slc15a2* | 26.7 | 0.015 | *Cidea* | -333.3 | 0.001 |
| *Ptk6* | 4.8 | 0.006 | *Ntrk1* | -25.0 | 0.021 |
| *Gm45425* | 4.3 | 0.016 | *Gm9922* | -25.0 | 0.046 |
| *Hamp2* | 4.0 | 0.012 | *C2cd4c* | -16.7 | 0.0001 |
| *Irx1* | 3.5 | 0.01 | *Cidec* | -16.7 | 0.007 |
| *Cyp2d12* | 3.5 | 0.024 | *Podxl2* | -14.3 | 0.002 |
| *Susd4* | 3.4 | 0.009 | *Plin4* | -12.5 | 0.002 |
| *Gm9803* | 3.2 | 0.001 | *Gal3st1* | -10.0 | 0.013 |
| *Slc22a7* | 3.2 | 0.009 | *Sdcbp2* | -10.0 | 0.048 |
| *Hcrtr2* | 3.1 | 0.03 | *Mmp24* | -10.0 | 0.002 |
| *C8b* | 3.1 | 0.018 | *Gm11695* | -9.1 | 0.044 |
| *Rdh16f2* | 2.9 | 0.003 | *Gm31600* | -8.3 | 0.0003 |
| *Cndp1* | 2.9 | 0.031 | *Fa2h* | -8.3 | 0.014 |
| *Vmn2r20* | 2.9 | 0.006 | *Ank1* | -8.3 | 0.046 |
| *Nat14* | 2.8 | 0.018 | *Trim6* | -8.3 | 0.009 |
| *Lrp2* | 2.8 | 0.01 | *Kbtbd11* | -8.3 | 0.025 |
| *Chic1* | 2.8 | 0.024 | *Gucy2c* | -8.3 | 0.025 |
| *Tsc22d3* | 2.8 | 0.027 | *Vsig8* | -7.7 | 0.048 |
| *Mup21* | 2.7 | 0.013 | *Gm20420* | -7.7 | 0.042 |
| *Slco1a1* | 2.7 | 0.049 | *Gm47352* | -7.7 | 0.01 |
| *Trhde* | 2.7 | 0.017 | *Ctse* | -7.7 | 0.006 |
| *B430119L08Rik* | 2.6 | 0.0002 | *Mthfd1l* | -7.1 | 0.034 |
| *Avpr1a* | 2.6 | 0.004 | *Bhlhb9* | -7.1 | 0.004 |
| *Ripply1* | 2.6 | 0.007 | *BC035947* | -7.1 | 0.042 |
| *Gm12718* | 2.6 | 0.028 | *Aqp7* | -7.1 | 0.04 |
| *Cyp2c54* | 2.6 | 0.04 | *Themis* | -6.3 | 0.006 |
| *Cyp2c29* | 2.5 | 0.007 | *Bmp8b* | -6.3 | 0.045 |
| *Wdr63* | 2.4 | 0.007 | *Gm13074* | -6.3 | 0.009 |
| *Adrb3* | 2.3 | 0.011 | *Mesp1* | -6.3 | 0.04 |
| *Gm40438* | 2.3 | 0.001 | *Tusc5* | -5.9 | 0.012 |
| *Apol9b* | 2.3 | 0.0001 | *Dcst2* | -5.3 | 0.001 |
| *Mup2* | 2.2 | 0.012 | *Endou* | -5.3 | 0.014 |
| *Sdr9c7* | 2.2 | 0.012 | *Rasl2-9* | -5.3 | 0.015 |
| *Slc10a2* | 2.2 | 0.011 | *Adgrg2* | -5.3 | 0.001 |
| *Cyp7b1* | 2.1 | 0.04 | *Cxcr2* | -5.3 | 0.036 |
| *Gna14* | 2.1 | 0.004 | *Osbpl3* | -5.3 | 0.014 |
| *2810425M01Rik* | 2.1 | 0.006 | *Dcstamp* | -5.3 | 0.002 |
| *Mup3* | 2.1 | 0.034 | *Zfp599* | -5.3 | 0.009 |
| *3110045C21Rik* | 2.1 | 0.017 | *Flrt3* | -5.0 | 0.033 |
| *Mafb* | 2.1 | 0.005 | *Mogat1* | -5.0 | 0.016 |
| *Apol9a* | 2.1 | 0.001 | *Gm9949* | -5.0 | 0.031 |
| *Gm15690* | 2.0 | 0.021 | *Slc13a4* | -4.8 | 0.009 |
| *Gm45470* | 2.0 | 0.011 | *Gm12454* | -4.8 | 0.029 |
| *Gm44421* | 2.0 | 0.002 | *Adamts16* | -4.5 | 0.017 |
| *Cyp2c37* | 2.0 | 0.025 | *Mesp2* | -4.5 | 0.005 |
| *Cyp1a2* | 2.0 | 0.0014 | *Lgals1* | -4.5 | 0.029 |
| *Gm17080* | 2.0 | 0.005 | *4930539E08Rik* | -4.5 | 0.0001 |
| *Slc17a8* | 2.0 | 0.008 | *Rad51b* | -4.3 | 0.026 |
| *Tat* | 2.0 | 0.041 | *Tubb2b* | -4.3 | 0.038 |
| *Gm10447* | 2.0 | 0.031 | *Acyp2* | -4.3 | 0.001 |
