# Supplementary material for: Hepatocyte-specific PPARγ Deletion Uncovers Role of an Antagonistic PPARγ–HNF4α Transcriptional Axis in Metabolic Dysfunction–Associated Steatotic Liver Disease Progression
Source: Am J Pathol. 2026 May 12;196(8):1563–80. doi: 10.1016/j.ajpath.2026.04.015 (PMC13494202; doi:10.1016/j.ajpath.2026.04.015)
Supplement: Supplementary file 12 [file mmc12.docx]

**Supplemental Table S2. Top 50 Genes That Were Up-regulated and Down-regulated in PPARγ -KO vs WT Mice on**

**FFD for 5 Months.**

| ***Up genes*** | ***KO/WT*** | ***P value*** | ***Down genes*** | ***KO/WT*** | ***P value*** |
| --- | --- | --- | --- | --- | --- |
| *Zbtb16* | 10.5 | 0.00012 | *Cidea* | -100.0 | 0.0052 |
| *Lrtm1* | 10.0 | 0.001 | *Sdcbp2* | -100.0 | 0.005 |
| *Cyp2b13* | 7.3 | 0.014 | *Gm47352* | -33.3 | 0.025 |
| *A4gnt* | 6.6 | 0.015 | *Fmr1nb* | -20.0 | 0.02 |
| *Chrna4* | 6.5 | 0.00004 | *Cxcl5* | -14.3 | 0.009 |
| *Slco1a1* | 6.2 | 0.049 | *Mup6* | -14.3 | 0.021 |
| *Arrdc2* | 5.7 | 0.03 | *Rufy4* | -12.5 | 0.002 |
| *Fkbp5* | 5.6 | 0.001 | *Ly6i* | -12.5 | 0.023 |
| *Atp2b2* | 5.5 | 0.001 | *Fabp3* | -11.1 | 0.045 |
| *Susd4* | 5.0 | 0.002 | *Tmem198* | -10.0 | 0.002 |
| *Rasl10b* | 5.0 | 0.006 | *Apol10b* | -10.0 | 0.021 |
| *Chic1* | 4.8 | 0.044 | *Gm31600* | -9.1 | 0.032 |
| *Ly6c1* | 4.5 | 0.037 | *Acod1* | -7.7 | 0.025 |
| *Lpin1* | 4.3 | 0.00021 | *Cxcl13* | -7.7 | 0.01 |
| *Sqle* | 4.1 | 0.014 | *Hamp* | -7.7 | 0.001 |
| *Clec2h* | 4.1 | 0.001 | *Ephb1* | -7.7 | 0.037 |
| *AC154912.1* | 4.1 | 0.008 | *Gm379* | -7.1 | 0.014 |
| *Cyp2a5* | 4.0 | 0.031 | *Kbtbd11* | -7.1 | 0.0003 |
| *1810046K07Rik* | 4.0 | 0.049 | *Cidec* | -6.7 | 1.0635E-06 |
| *Myom3* | 3.8 | 0.013 | *Gal3st1* | -6.7 | 0.001 |
| *Gna14* | 3.7 | 0.006 | *Bmp8b* | -6.7 | 0.003 |
| *C330021F23Rik* | 3.5 | 0.002 | *5031425F14Rik* | -6.3 | 0.023 |
| *Rec8* | 3.4 | 0.014 | *Fcer2a* | -6.3 | 0.009 |
| *Abcc12* | 3.4 | 0.023 | *Mup17* | -6.3 | 0.016 |
| *Gm11773* | 3.4 | 0.001 | *Mesp2* | -5.9 | 0.048 |
| *Blm* | 3.4 | 0.00035 | *Plin4* | -5.9 | 0.0002 |
| *Upp2* | 3.4 | 0.003 | *Chst3* | -5.9 | 0.014 |
| *Gm47140* | 3.3 | 0.016 | *Gprc5b* | -5.9 | 0.0004 |
| *Col15a1* | 3.2 | 0.004 | *Gm36161* | -5.6 | 0.016 |
| *Rbm24* | 3.2 | 0.014 | *Smad9* | -5.6 | 0.002 |
| *Gm10642* | 3.2 | 0.022 | *Fam167a* | -5.3 | 0.02 |
| *Ugt1a5* | 3.1 | 0.006 | *Olig1* | -5.3 | 0.003 |
| *Egfem1* | 3.1 | 0.038 | *A430078G23Rik* | -5.3 | 0.034 |
| *Pls1* | 3.0 | 0.005 | *Cd19* | -5.3 | 0.045 |
| *Ddit4* | 3.0 | 0.002 | *Hebp2* | -5.0 | 0.011 |
| *D830044D21Rik* | 3.0 | 0.022 | *Arsi* | -5.0 | 0.003 |
| *Mvd* | 3.0 | 0.0005 | *A530016L24Rik* | -5.0 | 0.033 |
| *Hmgcr* | 3.0 | 0.00083 | *Mkln1os* | -4.8 | 0.011 |
| *Cyp2b9* | 2.9 | 0.003 | *Fcmr* | -4.8 | 0.005 |
| *Usp2* | 2.9 | 0.046 | *F13a1* | -4.8 | 0.034 |
| *Cyp2c54* | 2.9 | 0.0003 | *Hr* | -4.5 | 0.0001 |
| *B430219N15Rik* | 2.9 | 0.007 | *Cd244a* | -4.3 | 0.032 |
| *Dusp14* | 2.9 | 0.00022 | *Gm47815* | -4.3 | 0.002 |
| *Mpped1* | 2.9 | 0.009 | *Cacnb1* | -4.3 | 0.037 |
| *Mas1* | 2.8 | 0.001 | *Bhlhb9* | -4.2 | 0.003 |
| *Acly* | 2.8 | 0.008 | *Mum1l1* | -4.2 | 0.027 |
| *Fdps* | 2.7 | 0.006 | *Slc25a31* | -4.2 | 0.021 |
| *Aqp4* | 2.7 | 0.002 | *Olr1* | -4.2 | 0.002 |
| *Tfrc* | 2.7 | 0.002 | *Ms4a1* | -4.2 | 0.003 |
| *Hkdc1* | 2.7 | 0.027 | *Dnah2* | -4.0 | 0.027 |
