# Supplementary material for: Hepatocyte-specific PPARγ Deletion Uncovers Role of an Antagonistic PPARγ–HNF4α Transcriptional Axis in Metabolic Dysfunction–Associated Steatotic Liver Disease Progression
Source: Am J Pathol. 2026 May 12;196(8):1563–80. doi: 10.1016/j.ajpath.2026.04.015 (PMC13494202; doi:10.1016/j.ajpath.2026.04.015)
Supplement: Supplementary file 13 [file mmc13.docx]

**Supplemental Table S3. Altered KEGG/Reactome Pathways in the 2-months Study: KO vs WT.**

| ***KEGG Pathway*** | ***Gene Count*** | ***P Value*** | ***Genes*** |
| --- | --- | --- | --- |
| Peroxisome | 14 | 8.15E-04 | *PECR, ABCD2, PEX19, PEX11A, IDH2, ACAA1B, ACAA1A, HMGCL, ACOX1, EHHADH, HACL1, DECR2, CRAT, PAOX* |
| Glycerophospholipid metabolism | 15 | 1.12E-03 | *PLA2G12A, CHKA, LPCAT1, PLA2G6, AGPAT2, PHOSPHO1, GPAT4, ETNK2, GPD2, GPD1, GPAT3, CHPT1, PLPP2, PLA1A, DGKH* |
| Fatty acid degradation | 9 | 6.71E-03 | *ALDH3A2, ADH4, ACOX1, EHHADH, ECI1, ACAA1B, CYP4A14, ACAA1A, ADH7* |
| Glycerolipid metabolism | 10 | 6.81E-03 | *ALDH3A2, GPAT4, MOGAT1, LPL, GPAT3, PLPP2, AGPAT2, AKR1B8, PNPLA2, DGKH* |
| PPAR signaling pathway | 12 | 8.83E-03 | *ACOX1, EHHADH, PLIN4, AQP7, LPL, PLIN2, ACAA1B, CYP4A14, CD36, SORBS1, ACAA1A, SLC27A4* |
| ECM-receptor interaction | 12 | 8.83E-03 | *COL1A1, TNXB, COL1A2, LAMB3, COL4A4, COL6A1, LAMA3, ITGA2B, COL6A3, LAMB1, CD36, FREM1* |
| Glycolysis / Gluconeogenesis | 9 | 2.86E-02 | *ALDH3A2, ADH4, PDHA1, ACSS2, PKLR, ALDH3B1, ADH7, HK2, HK1* |
| ***Reactome Pathway*** | ***Gene Count*** | ***P Value*** | ***Genes*** |
| Metabolism of lipids | 72 | 2.56E-08 | *PECR, SLC44A3, GAL3ST1, MOGAT1, ECI1, GDE1, LPCAT1, CYP3A11, PCTP, AKR1B8, NSDHL, CYP1B1, TSPO, ABCC3, ARSA, CERS5, PLA2G12A, CERS6, GPX1, GPX4, ACOT13, CYP4A14, CYP7B1, ALDH3A2, PHOSPHO1, ACOX1, EHHADH, GPD2, GPD1, ACOT1, PLPP2, ACOT3, PLA1A, VAC14, SLC22A5, AKR1D1, FITM1, ACAA1B, FITM2, HSD17B12, PLA2G6, AGPAT2, HSD11B1, HMGCL, GPAT4, ALDH3B1, DHRS7B, PSAP, GPAT3, HACL1, KDSR, DECR2, TMEM86B, OSBPL5, PLEKHA1, CHKA, SLC10A2, OSBPL3, SRD5A3, CIDEA, B3GALT4, PLEKHA4, SYNJ2, ACSF3, CIDEC, CYP4F14, FA2H, ETNK2, CYP1A2, CHPT1, CRAT, PNPLA2* |
| Fatty acid metabolism | 22 | 8.30E-04 | *PECR, SLC22A5, GPX1, GPX4, ECI1, ACOT13, ACAA1B, PCTP, CYP4A14, HSD17B12, ACSF3, CYP4F14, ALDH3A2, ACOX1, EHHADH, CYP1A2, ACOT1, CYP1B1, HACL1, DECR2, CRAT, ACOT3* |
| Collagen biosynthesis and modifying enzymes | 11 | 2.48E-03 | *COL1A1, COL3A1, BMP1, COL1A2, COL16A1, COL5A1, COL14A1, COL4A4, COL12A1, COL6A1, COL6A3* |
| Collagen formation | 11 | 1.21E-02 | *COL1A1, COL3A1, BMP1, COL1A2, COL16A1, COL5A1, COL14A1, COL4A4, COL12A1, COL6A1, COL6A3* |
| Collagen chain trimerization | 10 | 4.64E-04 | *COL1A1, COL3A1, COL1A2, COL16A1, COL5A1, COL14A1, COL4A4, COL12A1, COL6A1, COL6A3* |
| ECM proteoglycans | 10 | 1.80E-03 | *COL1A1, COL3A1, TNXB, COL1A2, COL5A1, COL6A1, ITGA2B, ITGAX, COL6A3, DCN* |
| Assembly of collagen fibrils and other multimeric structures | 10 | 2.08E-03 | *COL1A1, COL3A1, BMP1, COL1A2, COL5A1, COL14A1, COL4A4, COL12A1, COL6A1, COL6A3* |
| Collagen degradation | 10 | 2.76E-03 | *MMP12, COL1A1, COL3A1, MMP14, COL1A2, COL5A1, COL4A4, COL12A1, COL6A1, COL6A3* |
| Peroxisomal lipid metabolism | 8 | 6.88E-04 | *ALDH3A2, PECR, ACOX1, EHHADH, ACAA1B, HACL1, DECR2, CRAT* |
| Signaling by TGFBR3 | 5 | 4.14E-02 | *MMP14, TIMP2, PSEN2, INHA, TGFBR2* |
| Lipid particle organization | 4 | 3.42E-03 | *CIDEA, FITM1, FITM2, CIDEC* |
