# Supplementary material for: Hepatocyte-specific PPARγ Deletion Uncovers Role of an Antagonistic PPARγ–HNF4α Transcriptional Axis in Metabolic Dysfunction–Associated Steatotic Liver Disease Progression
Source: Am J Pathol. 2026 May 12;196(8):1563–80. doi: 10.1016/j.ajpath.2026.04.015 (PMC13494202; doi:10.1016/j.ajpath.2026.04.015)
Supplement: Supplementary file 14 [file mmc14.docx]

**Supplemental Table S4. Altered KEGG/Reactome Pathways in the 5-months Study: KO vs WT.**

| ***KEGG Pathway*** | ***Gene Count*** | ***P Value*** | ***Genes*** |
| --- | --- | --- | --- |
| Metabolic pathways | 225 | 1.81E-07 | *DMGDH, CERK, ACSM3, CYP3A11, CYP3A16, C1GALT1C1, NSDHL, PLCE1, MLYCD, GLUL, GAMT, ARSA, ENTPD1, ALG8, ACOD1, GBGT1, PISD, ADCY9, CYP2U1, ETHE1, MT-CO3, IDO2, ACOT4, GPLD1, MGST3, SHMT1, PLA2G6, PLA2G7, HACD4, ACMSD, HSD11B1, CYP2B9, LDHB, RDH11, INPP5D, PLCG2, HMOX1, ST3GAL5, HAO1, ASL, FDFT1, B3GALNT1, TMEM86B, SETDB2, ART2B, CYP26A1, SQLE, HAL, BHMT, AKR1B10, VNN1, PAH, FASN, CYP1A2, PDE3A, ECHDC1, RDH9, MOGAT1, SETD7, PIGP, GGTA1, AKR1B8, KHK, SMPD3, CYP2C37, SMPD2, CYP2B13, MECOM, CYP2B10, CD38, NANS, ARG2, ELOVL2, SPHK1, BHMT2, NME4, ELOVL6, RENBP, CAR5A, CAR5B, ITPKB, DPYD, ALDOC, ALDOB, PLCB2, GSTP3, IDUA, GSTP1, GCNT1, AKR1D1, ADCY4, HSD17B6, HSD17B7, ACNAT2, ACNAT1, ADCY6, PTGS1, GULO, EXTL1, MTHFD1L, CYP2C54, GPAT4, CYP2C50, GPAT3, CKB, CAR3, CAR2, CAR1, PKLR, CYP8B1, GCK, ETNPPL, GNMT, GATM, DHRS9, GPAM, GSTA3, ETNK2, GSTA2, LPCAT2, PNPLA3, GALNT12, ALDH1L1, MSMO1, SAT1, CNDP1, TM7SF2, HPGDS, CAR7, LARGE1, FADS1, DGAT2, GPT2, IPMK, PIPOX, ACYP2, PGD, ALDH3A2, ACLY, RDH16F2, PFKFB2, IDI1, PFKFB1, CYP4A12A, NPR2, GSTT3, GMPR, PLD4, ACACB, PLD3, ACACA, TKFC, AMDHD1, UAP1L1, PMVK, PRODH, FDPS, MGAM, SRD5A3, CYP2C29, CYP51, NAT8, TBXAS1, DHCR7, HPSE, GALK1, ALAS2, GAL3ST1, UGT2B1, TREH, PIK3CD, HK2, DHTKD1, GPI1, HK3, AOX3, DSE, PDE4B, UPP2, PLA2G12A, GPX1, GPX4, HMGCS1, URAD, PLA2G4A, CYP4A14, LSS, CYP2A5, DCT, ACOX1, ALDH1A2, HKDC1, ATP6V1B2, MVD, BLVRA, B4GALT5, AGPAT5, PDXK, ACSS3, ACSS2, MVK, ACAA1B, HSD17B12, ACAA1A, HMGCR, PPOX, HSD17B11, HSD17B10, AGPAT4, SELENBP1, UGP2, CHSY1, OXCT1, UGT1A5, GALNT6, RRM2, GALNT3, MMAB, NDUFA2, NAGK, MGAT4A, LPIN1, PFKM, LPIN2, LPIN3* |
| Steroid biosynthesis | 10 | 5.86E-05 | *SQLE, NSDHL, SOAT1, MSMO1, DHCR7, HSD17B7, LSS, CYP51, FDFT1, TM7SF2* |
| Inflammatory mediator regulation of TRP channels | 26 | 9.91E-04 | *PTGER4, CAMK2B, CYP4A12A, CAMK2D, SRC, ADCY4, PIK3CD, PLA2G6, ADCY6, CYP2C37, ASIC5, CYP2C54, PLCG2, CYP2C50, ASIC1, MAP2K6, PRKCB, PRKCD, TRPV2, PLA2G4A, CYP4A14, CYP2C29, ADCY9, IL1B, PRKCQ, PLCB2* |
| Glycerolipid metabolism | 16 | 1.11E-03 | *AGPAT5, DGAT2, MOGAT1, LPL, AKR1B8, AGPAT4, ALDH3A2, AKR1B10, TKFC, GPAM, GPAT4, PNPLA3, GPAT3, LPIN1, LPIN2, LPIN3* |
| NF-kappa B signaling pathway | 22 | 1.88E-03 | *LYN, CARD10, CD40, TICAM2, SYK, PRKCB, CXCL1, TNFRSF11A, NFKB2, ICAM1, PLAU, IL1B, CCL4, BTK, PLCG2, PRKCQ, LTB, BCL2A1D, CD14, TLR4, BCL2A1B, MYD88* |
| Cholesterol metabolism | 13 | 3.15E-03 | *ABCG8, MYLIP, ABCG5, SORT1, PCSK9, LPL, APOA4, NCEH1, SOAT1, NPC2, TSPO, LDLR, PLTP* |
| PPAR signaling pathway | 17 | 1.44E-02 | *CYP4A12A, HMGCS1, LPL, ACAA1B, CYP4A14, ACAA1A, SORBS1, CYP8B1, FABP3, ACOX1, FABP7, PLIN4, OLR1, PLIN2, PPARG, PPARA, PLTP* |
| ***Reactome Pathway*** | ***Gene Count*** | ***P Value*** | ***Genes*** |
| Metabolism of lipids | 124 | 3.19E-11 | *CERK, ECI1, CYP3A11, GDE1, MSMO1, CYP3A16, TM7SF2, NSDHL, HPGDS, MLYCD, FADS1, MFSD2A, ACOT9, ARSA, STARD4, DGAT2, SPNS2, ACOT11, ACOT13, SLC51B, ALDH3A2, ACLY, CYP2D22, CYP2U1, GPD2, GPD1, ACOT4, IDI1, CYP4A12A, PLA2G6, ACACB, ACACA, HACD4, HSD11B1, INPP5D, DHRS7B, PSAP, PMVK, ARSI, ST3GAL5, FDFT1, FDPS, B3GALNT1, TMEM86B, SLC10A1, SLC10A2, SRD5A3, CIDEA, CIDEC, CYP51, CYP4F14, SQLE, AKR1B10, CYP4F18, FASN, CYP1A2, ALB, TBXAS1, DHCR7, OSBPL1A, TNFAIP8, GAL3ST1, SLC44A2, MOGAT1, HILPDA, PIK3CD, PRKAG2, CROT, AKR1B8, SMPD3, SMPD2, TNFAIP8L1, TSPO, TNFAIP8L2, ABCD1, ABCC3, PLA2G12A, ABCC1, GPX1, HMGCS1, GPX4, ELOVL2, SPHK1, PLA2G4A, CYP4A14, ELOVL6, LSS, PITPNM1, ACOX1, MVD, PPARA, B4GALT5, AGPAT5, ACSS3, MVK, AKR1D1, FITM1, ACAA1B, HSD17B13, FITM2, HSD17B12, HMGCR, HSD17B7, HSD17B11, PTGS1, AGPAT4, GPAT4, OXCT1, GPAT3, PLEKHA1, CYP4B1, CYP8B1, ETNPPL, FABP3, GPAM, ETNK2, FABP7, LPCAT2, PNPLA3, THRSP, PNPLA5, LPIN1, LPIN2, LPIN3* |
| Neutrophil degranulation | 110 | 1.46E-10 | *ATP8A1, CLEC4N, GMFG, HP, IFI211, PYCARD, FCNA, STK10, LGALS3, PLAU, COTL1, C3AR1, SIRPA, PGLYRP1, ARSA, TICAM2, SLC11A1, PRKCD, CYBB, CYBA, ACLY, CLEC4D, RAB31, TYROBP, BIN2, FCGR4, S100A9, LYZ2, H2-M3, FPR1, GPR84, IQGAP1, FPR2, IQGAP2, CFP, PLAC8, RAP1B, SDCBP, DSN1, SERPINA3F, CYB5R3, PSAP, NCKAP1L, CD14, S100A11, GM5150, MGAM, ATP8B4, CD300C2, PTPRC, VNN1, SELL, SERPINB6A, LCN2, HPSE, ARHGAP9, ORM1, ITGAM, SLC44A2, ITGB2, CXCL1, ITGAL, SLC2A5, ORM3, ORM2, CTSS, GPI1, HK3, HEBP2, ADGRE5, OLR1, EAR2, CTSD, CD33, CTSC, CTSB, CD53, FCER1G, ANXA2, NFAM1, RHOG, ANO6, RHOF, FGR, NPC2, ALDOC, PLEKHO2, DOCK2, CD44, PDXK, PIRA2, CLEC12A, GSTP1, PTAFR, FGL2, PRCP, ACAA1B, PIRB, LILRA5, CST3, CD55, LAIR1, IFI204, IFI205, PTPN6, FCGR2B, FOLR2, CD68, FRK, HSPA1A* |
| Extracellular matrix organization | 45 | 1.37E-02 | *APP, COL15A1, ITGAM, SDC4, PCOLCE2, LAMA4, SDC2, COL12A1, ITGB2, SERPINE1, SDC3, ICAM2, TNC, LTBP2, ITGAL, NID1, THBS1, HAPLN1, CTSS, ICAM1, EFEMP2, EMILIN2, EMILIN1, ITGB7, TIMP1, CTSD, CTSB, TGFB1, ITGA4, TGFB3, LUM, DCN, ACTA2, MMP12, COL1A1, VCAN, COL3A1, COL1A2, BMP1, COL5A1, MMP15, COL5A3, COL5A2, COL8A1, CD44* |
| Fatty acid metabolism | 37 | 7.45E-05 | *CYP4A12A, ECI1, PRKAG2, ACAA1B, CROT, HSD17B12, ACACB, ACACA, HACD4, PTGS1, HPGDS, MLYCD, ABCD1, FADS1, ACOT9, ABCC1, GPX1, GPX4, ELOVL2, ACOT11, CYP4B1, ACOT13, PLA2G4A, ELOVL6, CYP4A14, CYP8B1, CYP4F14, ALDH3A2, ACLY, CYP4F18, CYP2U1, ACOX1, FASN, TBXAS1, CYP1A2, THRSP, ACOT4* |
| Metabolism of steroids | 32 | 1.30E-05 | *IDI1, MVK, AKR1D1, MSMO1, HSD17B12, HMGCR, HSD17B7, HSD17B11, AKR1B8, TM7SF2, HSD11B1, NSDHL, PMVK, TSPO, FDFT1, ABCC3, FDPS, STARD4, HMGCS1, SLC10A1, SLC10A2, SRD5A3, SLC51B, CYP8B1, LSS, CYP51, SQLE, AKR1B10, ALB, MVD, DHCR7, OSBPL1A* |
| Cholesterol biosynthesis | 16 | 9.00E-09 | *IDI1, FDPS, MVK, HMGCS1, MSMO1, HMGCR, HSD17B7, LSS, CYP51, TM7SF2, SQLE, NSDHL, PMVK, MVD, DHCR7, FDFT1* |
| Collagen degradation | 13 | 9.37E-03 | *COL15A1, COL12A1, MMP12, COL1A1, COL3A1, COL1A2, COL5A1, MMP15, COL5A3, COL5A2, COL8A1, CTSD, CTSB* |
| Assembly of collagen fibrils and other multimeric structures | 12 | 1.79E-02 | *COL1A1, COL15A1, COL3A1, BMP1, COL1A2, COL5A1, COL5A3, COL12A1, COL5A2, COL8A1, CTSS, CTSB* |
| Triglyceride metabolism | 10 | 6.39E-04 | *FABP3, DGAT2, GPAM, MOGAT1, GPD2, FABP7, PNPLA5, LPIN1, LPIN2, LPIN3* |
| Collagen chain trimerization | 9 | 7.47E-02 | *COL1A1, COL15A1, COL3A1, COL1A2, COL5A1, COL5A3, COL12A1, COL5A2, COL8A1* |
| Peroxisomal lipid metabolism | 7 | 6.87E-02 | *ALDH3A2, ACOX1, ACAA1B, MLYCD, CROT, ABCD1, ACOT4* |
| Lipid particle organization | 6 | 8.69E-05 | *HILPDA, CIDEA, FITM1, HSD17B13, FITM2, CIDEC* |
| Triglyceride biosynthesis | 6 | 9.61E-03 | *DGAT2, GPAM, MOGAT1, LPIN1, LPIN2, LPIN3* |
| LDL clearance | 6 | 4.99E-02 | *NCEH1, SOAT1, NPC2, PCSK9, LDLR, CES3A* |
