# Supplementary material for: Hepatocyte-specific PPARγ Deletion Uncovers Role of an Antagonistic PPARγ–HNF4α Transcriptional Axis in Metabolic Dysfunction–Associated Steatotic Liver Disease Progression
Source: Am J Pathol. 2026 May 12;196(8):1563–80. doi: 10.1016/j.ajpath.2026.04.015 (PMC13494202; doi:10.1016/j.ajpath.2026.04.015)
Supplement: Supplementary file 15 [file mmc15.docx]

**Supplemental Table S5. Pathways enriched in Common DEGs from the 2- and 5-Months Study: KO vs WT.**

| ***GO TERM_BP*** | ***Gene Count*** | ***P Value*** | ***Genes*** |
| --- | --- | --- | --- |
| lipid metabolic process | 24 | 1.58E-07 | *ARSA, MTMR1, IL1RN, ACSS2, GPX1, TMEM86B, PCX, MOGAT1, MGST3, BCKDHB, PLCL2, CIDEA, GDE1, ACOT13, LPL, HMGCL, ACOX1, GPAT4, GSTA3, HNF4A, SCAP, FLVCR2, SCLY, APOE* |
| inflammatory response | 22 | 5.61E-03 | *IL1RN, CAMK1D, TUSC2, RARRES2, PTAFR, STK39, CYBA, IL1RAP, CCL9, NAIP5, VNN1, DHRS7B, NINJ1, NDUFS4, AKT1, EPG5, CCL2, FAM3C, TLR5, MYD88, CCR2, SCYL1* |
| fatty acid metabolic process | 17 | 1.80E-07 | *CYB5A, LPL, ACAA1B, CYP4A14, CROT, ACAA1A, ALDH3A2, FA2H, GPAT4, NAAA, ACADM, ACOT6, ACAD11, DECR2, CRAT, SLC27A4, ACAD12* |
| response to oxidative stress | 16 | 2.72E-05 | *CAR3, SLC23A2, USP25, GPX1, GPX4, RBPMS, TAT, PRKCD, PRDX6, ATOX1, PRDX2, NAPRT, AKT1, MSRB3, APOE, GCLM* |
| cholesterol metabolic process | 13 | 2.41E-05 | *APLP2, HDLBP, PCTP, CLN6, APON, NSDHL, CES1C, CES1F, CYP1A2, APOF, SCAP, APOE, ABCG1* |
| xenobiotic metabolic process | 13 | 3.92E-05 | *CYP2J5, GSTP3, CYP2C29, CYP2C37, SULT1B1, CYP26A1, CYP2D12, NCEH1, CYP2D10, CYP2C54, GSTA3, HNF4A, CYP1A2* |
| extracellular matrix organization | 13 | 5.06E-03 | *COL27A1, IDUA, APLP2, FN1, HSD17B12, ADAMTS12, MMP12, COL1A1, VTN, LGALS3, COL3A1, COL1A2, MMP27* |
| TGFβ receptor signaling pathway | 11 | 4.34E-04 | *DUSP22, ACVRL1, CLDN5, COL3A1, COL1A2, BMP8B, LPXN, CCL2, LGALS9, GLG1, TGFBR2* |
| glucose homeostasis | 11 | 2.08E-02 | *GPR39, HNF4A, RPS6, ASPSCR1, AKT1, SLC37A4, FOXO3, ADRA1B, PFKM, MET, GCK* |
| regulation of inflammatory response | 9 | 5.63E-03 | *GRN, NLRP12, AREL1, IRF3, STK39, PIK3AP1, ADAMTS12, MYD88, CCR2* |
| ***Reactome Pathway*** | ***Gene Count*** | ***P Value*** | ***Genes*** |
| Metabolism of lipids | 67 | 3.22E-08 | *PECR, GAL3ST1, MOGAT1, ECI1, GDE1, CYP3A11, PRKAG2, PCTP, CROT, LCLAT1, AKR1B8, NSDHL, TNFAIP8L1, TSPO, ACADM, ACAD11, ABCC3, ARSA, PLA2G12A, GPX1, GPX4, ACOT13, CYP4A14, OCRL, ALDH3A2, ACOX2, ACOX1, EHHADH, GPD2, GPD1, MTMR1, MVK, AKR1D1, HSD17B4, FITM1, ACAA1B, FITM2, HSD17B12, HSD17B11, PLA2G6, AGPAT2, MTMR6, HSD11B1, HMGCL, GPAT4, AGMO, DHRS7B, PSAP, SCAP, GPAT3, SLC25A20, KDSR, DECR2, TMEM86B, PLEKHA1, SLC10A2, SRD5A3, CIDEA, CIDEC, CYP4F14, FA2H, ETNK2, ALB, CYP1A2, PI4KA, CRAT, PNPLA2* |
| Peroxisomal lipid metabolism | 10 | 5.83E-06 | *ALDH3A2, PECR, ACOX2, ACOX1, EHHADH, HSD17B4, ACAA1B, CROT, DECR2, CRATs* |
| Fatty acid metabolism | 23 | 8.16E-05 | *PECR, GPX1, GPX4, ECI1, HSD17B4, PRKAG2, ACOT13, ACAA1B, PCTP, CYP4A14, CROT, HSD17B12, CYP4F14, ALDH3A2, ACOX2, ACOX1, EHHADH, CYP1A2, ACADM, SLC25A20, ACAD11, DECR2, CRAT* |
| Lipid particle organization | 4 | 2.58E-03 | *CIDEA, FITM1, FITM2, CIDEC* |
| Metabolism of steroids | 15 | 6.89E-03 | *ABCC3, MVK, SLC10A2, SRD5A3, AKR1D1, HSD17B4, HSD17B12, HSD17B11, AKR1B8, HSD11B1, NSDHL, ACOX2, ALB, TSPO, SCAP* |
| ECM proteoglycans | 8 | 1.36E-02 | *COL1A1, ITGB1, VTN, COL3A1, COL1A2, COL5A1, FN1, DCN* |
| Assembly of collagen fibrils and other multimeric structures | 7 | 4.64E-02 | *COL1A1, COL3A1, BMP1, COL1A2, COL5A1, COL14A1, COL12A1* |
| Collagen chain trimerization | 6 | 6.27E-02 | *COL1A1, COL3A1, COL1A2, COL5A1, COL14A1, COL12A1* |
