# Supplementary material for: Hepatocyte-specific PPARγ Deletion Uncovers Role of an Antagonistic PPARγ–HNF4α Transcriptional Axis in Metabolic Dysfunction–Associated Steatotic Liver Disease Progression
Source: Am J Pathol. 2026 May 12;196(8):1563–80. doi: 10.1016/j.ajpath.2026.04.015 (PMC13494202; doi:10.1016/j.ajpath.2026.04.015)
Supplement: Supplementary file 16 [file mmc16.docx]

**Supplemental Table S6. List of genes altered downstream of TGF-β in PPARγ KO vs WT mice**

**fed FFD diet for 2 and 5 months.**

| ***Genes*** | ***KO/WT (2M)*** | ***Genes*** | ***KO/WT (5M)*** | ***Genes*** | ***KO/WT (5M)*** |
| --- | --- | --- | --- | --- | --- |
| *ADGRG2* | -5.3 | CXCL13 | -8.0 | *P2RY14* | -1.8 |
| *HK2* | -4.2 | HAMP | -7.6 | *COL1A2* | -1.8 |
| *ANXA2* | -3.4 | CHST3 | -5.8 | *TGM2* | -1.8 |
| *CCR1* | -3.3 | F13A1 | -4.7 | *POSTN* | -1.8 |
| *KDELR3* | -2.7 | HK2 | -3.9 | *C1QA* | -1.7 |
| *COL1A1* | -2.6 | ITGAM | -3.9 | *MSN* | -1.7 |
| *CCR2* | -2.4 | CD28 | -3.8 | *PITPNM1* | -1.7 |
| *COL3A1* | -2.4 | ADGRG2 | -3.7 | *COL8A1* | -1.7 |
| *TUBB2A* | -2.3 | SERPINE1 | -3.4 | *EGFR* | -1.7 |
| *COL1A2* | -2.3 | TPM2 | -3.1 | *RAB31* | -1.7 |
| *NT5E* | -2.3 | RASGRP1 | -3.0 | *G6PC1* | -1.7 |
| *ADAMTS12* | -2.2 | PTAFR | -3.0 | *TNFRSF12A* | -1.7 |
| *HBEGF* | -2.2 | CXCR6 | -2.9 | *SLC25A4* | -1.6 |
| *ANXA5* | -2.1 | IL1B | -2.8 | *ZEB2* | -1.6 |
| *INHA* | -2.1 | S1PR4 | -2.8 | *Cxcl1* | -1.6 |
| *CDKN1C* | -2.1 | LCN2 | -2.7 | *TGFB1* | -1.6 |
| *PTAFR* | -2.0 | TNC | -2.7 | *CTPS1* | -1.6 |
| *COL12A1* | -2.0 | ACTA2 | -2.7 | *COL12A1* | -1.6 |
| *TPM2* | -1.9 | LTBP2 | -2.6 | *AXL* | -1.6 |
| *EDN1* | -1.9 | VCAN | -2.6 | *MRC1* | -1.6 |
| *COL6A3* | -1.8 | CCL2 | -2.5 | *PTPN6* | -1.6 |
| *COL5A1* | -1.8 | RUNX3 | -2.4 | *KDELR3* | -1.6 |
| *PDE4D* | -1.8 | IL1RN | -2.4 | *ACTN1* | -1.5 |
| *IL1RN* | -1.8 | PLAU | -2.3 | *NEDD9* | -1.5 |
| *COL6A1* | -1.8 | S100A11 | -2.3 | *NRP1* | -1.5 |
| *TXNIP* | -1.7 | CD44 | -2.3 | *CSRP1* | -1.5 |
| *BCL2L11* | -1.7 | ABCG1 | -2.2 | *INPP5D* | -1.5 |
| *CDKN2C* | -1.7 | CCNB1 | -2.2 | *SOD3* | -1.5 |
| *LAMB3* | -1.7 | PCOLCE2 | -2.2 | *RNF152* | -1.5 |
| *PRDM1* | -1.6 | SLC51B | -2.2 | *CSRP2* | -1.5 |
| *ELMO1* | -1.6 | Cdkn2a | -2.2 | *GPD1* | -1.4 |
| *JUNB* | -1.6 | ACVRL1 | -2.2 | *PLXNC1* | -1.4 |
| *DOK1* | -1.6 | Cxcl10 | -2.2 | *CBFB* | -1.4 |
| *PDGFRA* | -1.6 | CCL24 | -2.2 | *DAB2* | -1.4 |
| *SLC25A4* | -1.6 | PIK3CD | -2.2 | *CSF1* | -1.4 |
| *GPD1* | -1.6 | Ccl7 | -2.1 | *PALS2* | -1.4 |
| *TGFBR2* | -1.6 | TIMP1 | -2.1 | *COL5A1* | -1.4 |
| *COL16A1* | -1.6 | CTSS | -2.1 | *TUBB2A* | -1.4 |
| *CYBA* | -1.6 | PROCR | -2.1 | *CTSD* | -1.4 |
| *ADAMTS10* | -1.6 | TNFAIP8 | -2.1 | *SGK1* | -1.4 |
| *IL33* | -1.6 | NLRP3 | -2.1 | *VASP* | -1.4 |
| *P2RY14* | -1.6 | SPI1 | -2.1 | *ADAMTS12* | -1.4 |
| *MYO1C* | -1.5 | CD74 | -2.1 | *GJA1* | -1.4 |
| *SLC2A1* | -1.5 | COL1A1 | -2.0 | *GCNT1* | -1.4 |
| *ARHGEF2* | -1.5 | ITGB2 | -2.0 | *APP* | -1.4 |
| *CCL2* | -1.5 | PLA2G4A | -2.0 | *ANXA5* | -1.4 |
| *ABCG1* | -1.5 | TLR4 | -2.0 | *Cst3* | -1.4 |
| *TGFBI* | -1.5 | IL1A | -2.0 | *VEGFC* | -1.3 |
| *FUT8* | -1.5 | CXCR4 | -2.0 | *HSD17B10* | -1.3 |
| *CD274* | -1.4 | S100A6 | -2.0 | *EMILIN1* | -1.3 |
| *SNAI1* | -1.4 | PDLIM4 | -2.0 | *COTL1* | -1.3 |
| *PDGFB* | -1.4 | CHST11 | -2.0 | *IGFBP3* | -1.3 |
| *TAX1BP3* | -1.4 | HSPB1 | -2.0 | *LSM6* | -1.3 |
| *DKK3* | -1.4 | COL3A1 | -2.0 | *CEBPD* | -1.3 |
| *MMP14* | -1.4 | CCR2 | -2.0 | *MYL6* | -1.3 |
| *PRKCA* | -1.4 | CYBA | -2.0 | *CLU* | -1.3 |
| *ACVRL1* | -1.4 | CD72 | -2.0 | *CTSB* | -1.3 |
| *TGFA* | -1.4 | CD14 | -1.9 | *TSPAN7* | -1.3 |
| *SMTN* | -1.4 | C1QB | -1.9 | *H2AX* | -1.3 |
| *CSRP2* | -1.4 | C1QC | -1.9 | *CFH* | -1.3 |
| *LEMD3* | -1.4 | FILIP1L | -1.9 | *NOTUM* | 1.3 |
| *WWTR1* | -1.4 | ANXA2 | -1.9 | *Selenbp1/2* | 1.3 |
| *PALS2* | -1.3 | THBS1 | -1.9 | *AQP8* | 1.3 |
| *MYL12B* | -1.3 | SEMA3B | -1.9 | *SRC* | 1.3 |
| *ZEB2* | -1.3 | HDAC9 | -1.9 | *GLUL* | 1.4 |
| *HK1* | -1.3 | VIM | -1.9 | *OCLN* | 1.4 |
| *CORO1C* | -1.3 | TNFRSF11A | -1.9 | *HMGCS1* | 1.4 |
| *Ceacam1/2* | 1.3 | DOCK2 | -1.9 | *CCNF* | 1.4 |
| *LSR* | 1.3 | NCF2 | -1.9 | *ALB* | 1.5 |
| *MLXIPL* | 1.3 | HMOX1 | -1.9 | *LDLR* | 1.6 |
| *CRP* | 1.3 | CSF1R | -1.9 | *Ces2c* | 1.6 |
| *ITIH3* | 1.3 | C3AR1 | -1.8 | *KCNN2* | 1.6 |
| *ASGR2* | 1.3 | P2RY6 | -1.8 | *STARD4* | 1.6 |
| *CCNF* | 1.4 | NUCB2 | -1.8 | *TERT* | 1.8 |
| *KLF15* | 1.5 | CYBB | -1.8 | *CYP51A1* | 1.8 |
| *Apon* | 1.5 | HBEGF | -1.8 | *RDH11* | 1.8 |
| *DNMT3B* | 1.6 | TNFRSF11B | -1.8 | *RRM2* | 1.9 |
| *Serpina1a* | 2.0 | ME2 | -1.8 | *HMGCR* | 3.0 |
| *GNA14* | 2.1 | PTGS1 | -1.8 | *PPARGC1A* | 2.4 |
