# Supplementary material for: Hepatocyte-specific PPARγ Deletion Uncovers Role of an Antagonistic PPARγ–HNF4α Transcriptional Axis in Metabolic Dysfunction–Associated Steatotic Liver Disease Progression
Source: Am J Pathol. 2026 May 12;196(8):1563–80. doi: 10.1016/j.ajpath.2026.04.015 (PMC13494202; doi:10.1016/j.ajpath.2026.04.015)
Supplement: Supplementary file 17 [file mmc17.docx]

**Supplemental Table S7. List of genes altered downstream of HNF4α in PPARγ-KO vs WT mice**

**fed FFD diet for 2 and 5 months.**

| ***Genes*** | ***KO/WT (2M)*** | ***Genes*** | ***KO/WT (5M)*** | ***Genes*** | ***KO/WT (5M)*** |
| --- | --- | --- | --- | --- | --- |
| *Slco1a1* | 2.7 | *Slco1a1* | 6.2 | *LGR5* | -1.5 |
| *CYP7B1* | 2.1 | *Clec2e/2h* | 4.1 | *MYC* | -1.5 |
| *TAT* | 2.0 | *Gsta1* | 2.5 | *KRT18* | -1.5 |
| *Serpina1a* | 2.0 | *ACSS2* | 2.4 | *CCND1* | -1.5 |
| *ACMSD* | 1.9 | *PPARGC1A* | 2.4 | *AP1S2* | -1.7 |
| *F11* | 1.5 | *CYP8B1* | 2.3 | *MSN* | -1.7 |
| *Apon* | 1.5 | *LAD1* | 2.2 | *LORICRIN* | -1.8 |
| *KLF15* | 1.5 | *TM7SF2* | 2.1 | *CKB* | -1.8 |
| *IL1RAP* | 1.5 | *NR0B2* | 2.1 | *HBEGF* | -1.8 |
| *ACSS2* | 1.4 | *PKLR* | 2.1 | *HDAC9* | -1.9 |
| *PKLR* | 1.4 | *SLC10A1* | 2.0 | *PLA2G4A* | -2.0 |
| *PNP* | 1.4 | *Cyp2b10* | 1.8 | *CCNB1* | -2.2 |
| *ITIH3* | 1.3 | *PRODH* | 1.7 | *SYBU* | -2.4 |
| *CRP* | 1.3 | *Ces2c* | 1.6 | *LPL* | -2.7 |
| *MLXIPL* | 1.3 | *LDLR* | 1.6 | *TNC* | -2.7 |
| *Ceacam1/Ceacam2* | 1.3 | *ALB* | 1.5 | *HAMP* | -7.6 |
| *PSAP* | -1.3 | *NR1I3* | 1.5 | *ACOD1* | -8.0 |
| *TLN2* | -1.3 | *APOA4* | 1.4 |  |  |
| *FHOD1* | -1.4 | *F11* | 1.4 |  |  |
| *FUT8* | -1.5 | *ALDOB* | 1.4 |  |  |
| *SLC2A1* | -1.5 | *GPI* | 1.4 |  |  |
| *KRT18* | -1.6 | *ZFP36* | 1.4 |  |  |
| *KRT8* | -1.6 | *ELOVL2* | 1.4 |  |  |
| *LEPR* | -1.6 | *ANKRD13B* | 1.3 |  |  |
| *ELMO1* | -1.6 | *AQP8* | 1.3 |  |  |
| *PRDM1* | -1.6 | *SLC20A1* | 1.3 |  |  |
| *LPL* | -1.7 | *ACMSD* | 1.3 |  |  |
| *LAMB3* | -1.7 | *SLC25A1* | 1.3 |  |  |
| *EID1* | -1.7 | *PPARA* | 1.3 |  |  |
| *CD36* | -1.9 | *KRT8* | -1.4 |  |  |
| *ABCD2* | -1.9 | *APP* | -1.4 |  |  |
| *PRDM16* | -2.0 | *RPS12* | -1.4 |  |  |
| *HBEGF* | -2.2 | *PSAP* | -1.4 |  |  |
| *ACOT1* | -3.9 | *TLN2* | -1.4 |  |  |
| *TOX* | -4.0 | *RNF152* | -1.5 |  |  |
