# Supplementary material for: Hepatocyte-specific PPARγ Deletion Uncovers Role of an Antagonistic PPARγ–HNF4α Transcriptional Axis in Metabolic Dysfunction–Associated Steatotic Liver Disease Progression
Source: Am J Pathol. 2026 May 12;196(8):1563–80. doi: 10.1016/j.ajpath.2026.04.015 (PMC13494202; doi:10.1016/j.ajpath.2026.04.015)
Supplement: Supplementary file 18 [file mmc18.docx]

**Supplemental Table S8. List of DEGs common in 2- and 5-month studies linked to both PPAR and HNF4α binding sites (shown as purple overlap in Figure8J).**

*Alb, Apoe, Hp, Mgst1, Bhmt, Acox1, Acaa1b, Car3, Ces1c, Tat, Fn1, Gnmt, Plin2, Aldh3a2, Cyb5a, Aldh1l1, Reep6, Pcx, Trf, Sec14l2, Crot, Slc25a47, Cyp2d10, Serping1, Hsd17b4, Prdx6, Krt8, Mettl7b, Hnf4a, Ghr, Lgals9, Apof, Gpx4, Ehhadh, Haao, Serpinf1, Pklr, Acadm, Slc25a39, Gpd1, Dpyd, Gck, Serpina3m, Dmgdh, Abcb4, Suclg2, Apoc4, Cd36, Iqgap2, Apon, Acad11, Nudt4, Rarres2, Ak3, Krt18, Aldh9a1, Cd81, Gclm, Cpn2, Cyp1a2, Pabpc1, Ptprf, Slc25a10, Agpat2, Etnk2, Edem1, Cdc42, Ppp2r5a, Acox2, Slc25a23, Acaa1a, Myo1b, Psen2, Grn, Decr2, Shmt2, Kynu, Ptp4a2, Gpat4, Ddah1, Slc25a13, Slc26a1, Akr1d1, Gckr, Bbox1, Slc31a1, Slc9a3r1, Mthfd1, C2cd2, Slc37a4, Adi1, Serpina12, Ppm1a, Dio1, Tst, Shmt1, Eci1, Ninj1, Gpld1, Slc39a14, Tsc22d3, Pecr, Abhd2, Tns1, Cyp4f14, Hs6st1, Chchd10, Rassf3, Slco2b1, Asgr2, Hgfac, Fbxo3, Ptprd, Pgrmc2, Atp9a, C1rl, Pbld2, Pexpli19, Slc29a1, Lrpprc, Ahcyl1, Col14a1, Naprt, Cox7a2l, Pctp, Pex11a, Wbp2, Hp1bp3, Scly, Lrrc3, Bmp1, Cd59a, Galk1, Tmem120a, Apol7a, Rnf149, Usp4, Enpep, Camk1d, Ergic1, Met, Cldn1, 5031439G07Rik, Spp2, Pgpep1, Crat, Cidec, Cmtm4, Slc10a2, Scap, Cdc42ep4, Fam214a, Timd2, Paqr7, Ido2, Pik3ap1, Aspscr1, Ankrd40, Fgf1, Foxo3, Myo1c, Csk, Zfp36l2, Gpd2, Anapc5, Pla2g6, Grb14, Adra1b, Aspdh, Cmas, Rmdn2, Dusp3, Acot13, Gphn, Gmfb, Hes6, Thnsl2, Lclat1, Tlcd2, Slc41a2, Hyal2, Lgr4, Nceh1, Nln, Ccl9, Arel1, Creld1, Pyroxd2, Emg1, Pdzd8, Cyp26a1, Dennd4a, Rbpms, Car5a, Arl4a, Palmd, Tedc2, Tbc1d16, Dhrs7b, Zfp655, Mast3, Klhdc7a, Spred1, Lsm4, Abcb10, Ankrd33b, Abhd6, Clstn3, Col27a1, Tnfaip8l1, Sorbs1, Mcc, Ppp2r1b, Fcna, Tmem260, Spata2l, Mgst3, Prkag2, Gstp3, Fam124a, Csrp2, Emc8, Slc3a1, Slc35f6, Slc46a3, Cnp, Adap2, Nup54, Stard13, Arhgap26, Aqp4, Enc1, Fam47e, Wdr73, Nlrp12, Pla2g12a, Stat5a, Nat8, Mycl, Il1rn, Urad, Camk2d, Slc25a33, Klf11, Tmem186, Rtn4rl1, S1pr2, Rnf186, Slc22a7, Acmsd, Mnt, Carmil1, Gpat3, Ccdc162, Rdh9, Avpr1a, Acvrl1, Cldn5, L3mbtl3, Fign, Gna14, Zfp141, Dusp10, Flvcr2, Tc2n, Gpr39, Ten1, Fam89a, Coro2a, and Jpx.*
